# Evaluating the role of synanthropic filth flies in the transmission of zoonotic parasites: field and laboratory evidence from different animal rearing sites in upper Egypt with focus on *Cryptosporidium* spp.

**DOI:** 10.1186/s12917-025-04627-w

**Published:** 2025-03-20

**Authors:** Omaima Ragab AbdAllah, Refaat M. Gabre, Sara Abdelaal Mohammed, Ahmed Mohamed Korayem, Hala E. Hussein, Alzahraa Abdelraouf Ahmad

**Affiliations:** 1 Entomology branch at Department of Zoology/Entomology, Faculty of Science, Assiut University, Assiut, 71515 Egypt; 2 Department of Biotechnology, Faculty of Science, Cairo University, Cairo, 12613 Egypt; 3 Department of Veterinary Parasitology, Faculty of Veterinary Medicine, Assiut University, Assiut, 71515 Egypt; 4 Department of Biology, College of Arts and Sciences, Gonzaga University, Spokane, WA USA; 5 Department of Medical Parasitology, Faculty of Medicine, Assiut University, Assiut, 71515 Egypt

**Keywords:** Filth flies, Animal farms, Upper Egypt, Mechanical vector, Zoonotic parasite infections, *Cryptosporidium*, Nested PCR, Sequencing

## Abstract

**Background:**

Synanthropic filth flies thrive in human and animal habitats, posing health risks through the transmission of infectious agents. They breed on organic waste, including animal feces, making them carriers of various pathogens. In Egypt, where livestock farming is common and poor sanitation, these flies may contribute to zoonotic disease transmission. The current study investigates parasitic infections in filth flies from three livestock farms in Assiut Governorate, Upper Egypt, highlighting their role as vectors for zoonotic infections, particularly *Cryptosporidium*, via morphological and molecular tools.

**Methods:**

A total of 12,749 flies were collected from the study sites via sweep nets. After taxonomic identification, the flies were examined microscopically for parasites using various concentration and staining techniques. Positive samples were further confirmed for infections, particularly for *Cryptosporidium* parasites, via nested PCR and sequence analysis targeting the COWP and SSU rRNA genes.

**Results:**

This study revealed the presence of several fly species from seven dipteran families, particularly the family Muscidae, primarily *Musca domestica*, which presented a high parasite infestation rate of 96.6%. This study revealed a high prevalence of various protozoans and helminths in the collected flies*. Cryptosporidium* was the most prevalent parasite (64.4–100%), infecting all fly species. *Entamoeba* and *Balantidium* were also significant, especially in *M. domestica* (22.6–90.1%, 8.9–100%), *Fannia canicularis* (10.5–74.4%, 44.2–88.2%), and *Borborillus vitripennis* (11.1–50%, 37.2–91.4%). *Giardia, Trichuris, and Trichostrongylidae* had low to moderate prevalence in multiple fly species*.* Mites are commonly detected on fly exoskeletons, with high infestation rates observed in *Musca domestica* (77–100%) and *Physiphora alceae* (66.7–100%). The present study also reported sporadic infections with *Trichomonas*, *Toxocara vitulorum*, and pseudoscorpions, along with notable midge larval infestations (52.1%), mainly at site B. Parasitic infections were highest in autumn and spring, with the lowest rates in winter. Molecular identification confirmed the presence of the zoonotic species *Cryptosporidium parvum* and *Cladotanytarsus gedanicus*.

**Conclusion:**

This study revealed that zoonotic parasites exist in flies and pose potential risks when they are found near humans. *Cryptosporidium parvum* is the prevalent parasite causing diarrhea outbreaks in animals. This is the first genetic evidence of *Cladotanytarsus gedanicus* midge from Upper Egypt.

**Supplementary Information:**

The online version contains supplementary material available at 10.1186/s12917-025-04627-w.

## Introduction

Synanthropic filth flies represent nonbiting species that have evolved to thrive in areas of human and animal activities, such as food markets, restaurants, and poultry and livestock farms. They are classified within families such as Muscidae (house flies), Calliphoridae (blowflies), and Sarcophagidae (flesh flies). These flies are frequently encountered in unsanitary conditions, where they breed in organic waste, refuse, and fecal material. They are associated with the mechanical transmission of parasites, fungi, bacteria, and viruses [1–5]. Since they interact with human food, domestic animals, and waste, they transmit various diseases [6].

Houseflies (*Musca domestica*) are widespread pests and are the main synanthropic flies in agricultural settings and residential areas. Controlling house fly populations is a significant challenge for livestock farming because of their ideal breeding and feeding conditions on farms. They can ingest and carry pathogens on their bodies, transmitting protozoan cysts, such as those of *Entamoeba*, *Cryptosporidium*, and *Giardia*, which are recognized for causing gastrointestinal infections. Additionally, houseflies can transport larger parasites, such as helminth eggs, including *Taenia*, *Dipylidium*, *Diphyllobothrium*, *Ancylostoma*, *Enterobius*, *Trichiuris* and *Ascaris,* on their exoskeletons [7].

Flies transmit pathogens mechanically by picking them up from fecal matter and transferring them to other surfaces or food when they land [8]. During the winter, barns are particularly favorable locations for the breeding and overwintering of synanthropic flies [9]. The viscosity of feces makes them more vulnerable to synanthropic flies, such as houseflies, which transmit infectious pathogens to other surfaces [7]. In addition, the cyst stages of infectious intestinal protozoans can remain infectious after passing through a fly's digestive system. This may lead to surface contamination when the fly settles and discharges feces, transmitting human infections via deposition and regurgitation. Furthermore, airborne particles from fly electrocutting traps might transfer human disease agents, increasing the risk of infection [10].

Several studies have been conducted in Egypt to investigate the prevalence of filth flies around human and animal dwellings. For example, the dominant fly species in Suez Province, located in Lower Egypt, include *Chrysomya megacephala, Lucilia cuprina,* and *Musca domestica* [11]. However, dipterous fly species belonging to the families Calliphoridae, Muscidae, Otitidae, Piophilidae, Sarcophagidae, Sphaeroceridae, Milichiidae, Chloropidae, Drosophilidae, Sepsidae and Phoridae have been recorded in Menoufia Governorate in the Nile Delta [12]. Moreover, houseflies from the Muscidae family are the most prevalent species found in Sohag Governorate, Upper Egypt [13].

Synanthropic flies, particularly the housefly *M. domestica,* have been reported as pathogen carriers that cause infectious diarrhea. Diarrhea likely leads to dehydration and may cause death and other threatening conditions in humans and animals [14, 15]. The incidence of diarrhea increases during periods of high fly density and decreases as the density of houseflies decreases [16, 17].

In Egypt, intestinal parasites are regarded as a major public health issue and are a primary contributor to diarrheal diseases, with prevalence rates reaching as high as 61% [18]. Research shows elevated levels of protozoan and helminth infections within the general population, particularly among vulnerable groups such as individuals with decreased immunity and chronic diarrhea[19]. *Cryptosporidium* spp.*, Entamoeba histolytica, and Giardia intestinalis* are the most common protozoa responsible for causing diarrheal illnesses in Egypt and around the world [18]. *E. histolytica* has been reported to be highly prevalent in patients experiencing diarrhea in Egypt [20]. *G. intestinalis* frequently causes diarrhea in children, with prevalence rates ranging from 11% to 15.4% [21, 22]. *Cryptosporidium* can also infect humans and animals, leading to severe diarrhea, particularly in immunocompromised individuals [23]. In addition, intestinal nematode infections, such as those caused by *Trichuris* and *Toxocara* species, are relatively common and can lead to malnutrition and cognitive issues. The prevalence of these infections is linked to poor sanitation, inadequate water supply, and low socioeconomic status, which promote transmission through contaminated food and water[18]*.*

Cryptosporidiosis and giardiasis are zoonotic diseases with complex epidemiology due to various reservoir hosts. These parasites can survive in harsh environmental conditions and are transmitted through the ingestion of oocysts or cysts from infected sources, often through contaminated food or water [24]. Numerous outbreaks have been reported worldwide [25–27], and flies are considered a significant risk factor for the spread of these parasites, particularly in rural livestock areas [24, 28]. Furthermore, the transmission of cryptosporidiosis from farm animals to farmers has been confirmed to pose economic risks and highlights the danger of zoonotic diseases [29].

Several studies have investigated fly-associated transmission of zoonotic parasites, particularly *Cryptosporidium,* in human settings and from animal farms [24, 30–33]. However, very few studies have been conducted on the potential role of flies as mechanical vectors of parasites in Egypt [34]. While morphological observation of eggs and cysts is a common identification method, it can lead to inaccuracies due to species similarities [28]. Therefore, molecular techniques such as conventional and nested PCR offer increased specificity and sensitivity at a low cost. The use of genetic markers such as the COWP and SSU rRNA genes for *Cryptosporidium* spp. and various loci for *Giardia lamblia* can increase our understanding of these parasites and their transmission, facilitating better monitoring and management strategies [35, 36].

The present study aims to determine the potential role of various species of synanthropic filth flies as carriers of zoonotic parasites at three different animal-rearing sites in Assiut Governorate, Upper Egypt, with a particular emphasis on *Cryptosporidium* infection via morphological tools and molecular studies.

## Materials and methods

### Study sites

**Fig. 1:**
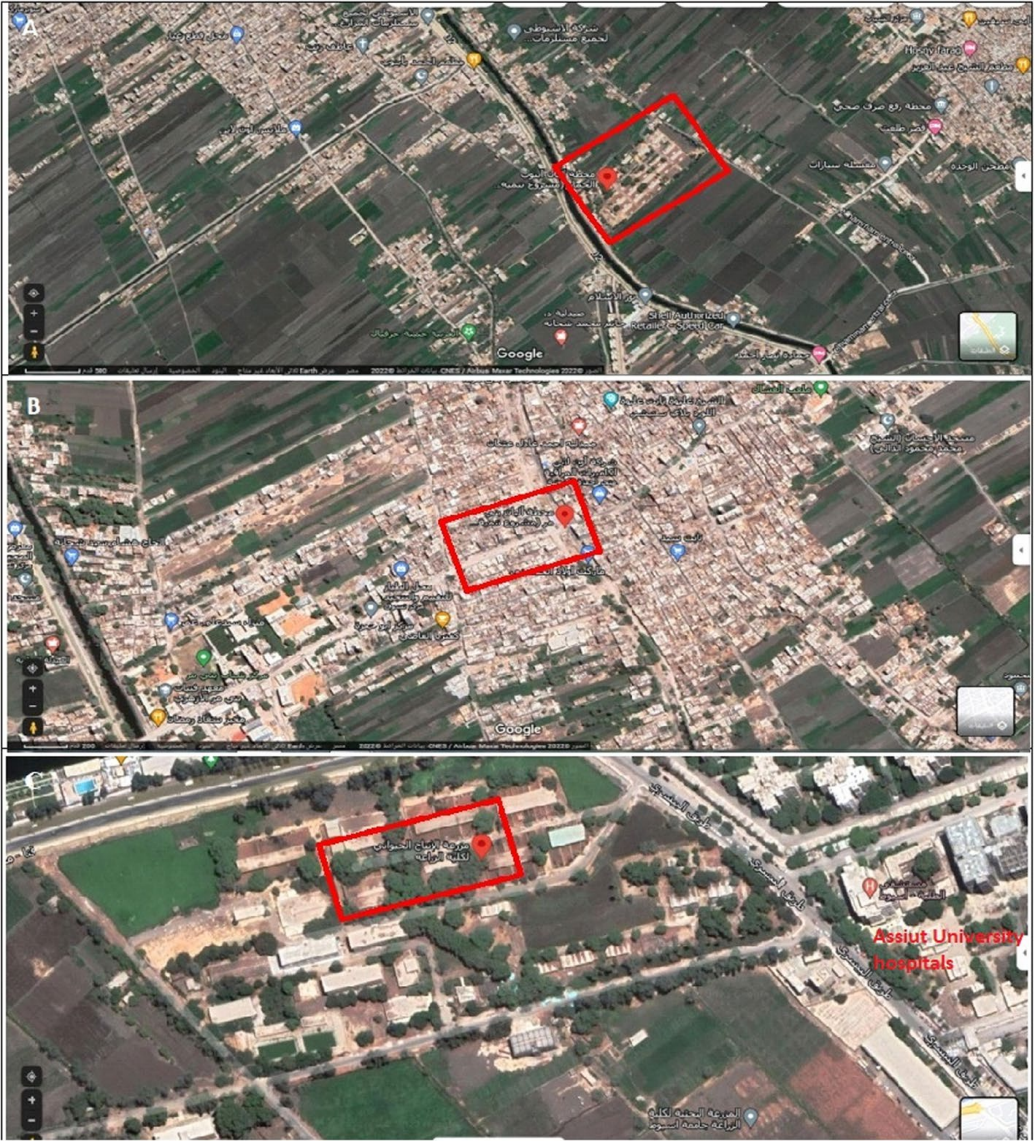
A satellite image from Google Earth showing the studied animal sites (**A**, **B**, and **C**)

This research was carried out at animal-rearing sites in Assiut Governorate, Upper Egypt (27.252°N31.01°E), located approximately 375 km south of Cairo, over one year from July 2020 to June 2021. The research focused on three distinct animal-rearing sites, selected primarily on the basis of their proximity to human settings. Site A is located in El-Hammam village, Abnoub district, approximately 9.01 km northeast of Assiut city, where *Bos taurus* (Holstein Friesian) of different ages are]reared; Site B is located in Bani Murr village, El-Fath district, approximately 4.37 km northeast of Assiut city, which houses Egyptian buffaloes of varying ages; and site C is located approximately 2.73 km northwest of Assiut city and adjacent to Assiut University hospitals, where Holstein Friesians, Egyptian cows, and buffaloes are raised (Fig. 1). All samples were collected from traditional smallholder farms all of which are characterized by small-scale, traditional animal-rearing practices. These farms typically have limited sanitation infrastructure, and higher exposure to organic waste.

### Collection of fly samples

A sweep net was used to collect a total of 12,749 flies belonging to seven dipteran families near fresh animal feces, which were then transferred into sterile, labeled plastic jars and taken to the entomology laboratory at the Faculty of Science, Assiut University. To immobilize the flies, jars were placed in a freezer at −20 °C. The flies were taxonomically identified via standard morphological keys [37, 38]. Afterwards, the flies were grouped into pools (each containing 7–10 flies) and placed in 1.5 ml Eppendorf tubes filled with 1 ml of phosphate-buffered saline (PBS). They were then thoroughly washed via a vortex to dislodge eggs and cysts of parasites that are attached to the insect's body and then centrifuged at 3000 rpm for 5 min to ensure the recovery of the particles derived from the exoskeletons of the flies in the sedimented debris [39].

### Parasite isolation and identification

1. Diagnosis of helminth eggs/larvae and protozoan cysts.
  Morphological diagnosis

To enhance parasite detection, the sample concentration was done. Initially, a portion of the fly wash was subjected to a formalin-ether sedimentation technique to concentrate larger parasites, such as helminth eggs and larvae [40]. Another portion of the fly wash was subjected to a floatation technique to isolate *Cryptosporidium* oocysts [41]. Parasites were morphologically identified based on taxonomic identification keys via light microscopy at various magnifications (10X, 40X and 100X) via various staining techniques, including Lugol's iodine, methylene blue, and modified Ziehl–Neelsen stain [42, 43].

- Molecular diagnosis of Cryptosporidium via a nested PCR assay

*Cryptosporidium-*positive samples were subjected to multiple cycles of freeze-thawing to disrupt the oocyst wall and facilitate subsequent DNA extraction. Briefly*,* microscopically positive elutes were subjected to five cycles of freezing in liquid nitrogen and thawing to 98 °C for an equal amount of time to disrupt the *Cryptosporidium* oocyst wall [44]. The genomic DNA of *Cryptosporidium* was subsequently extracted via a QIAamp® DNA Mini Kit from QIAGEN (Germany) (Cat. no. 51504). Nested PCR was performed to target two genes, the *Cryptosporidium* oocyst wall protein (*COWP*) gene and the small subunit ribosomal RNA (*SSUrRNA)* gene, according to previous studies [45, 46].

- Diagnosis of isolated nematode larvae by scanning electron microscopy (SEM).

Nematode larvae isolated from fly washes were processed via SEM to confirm the diagnosis. Samples of nematode larvae were dehydrated in increasing grades of ethanol and then stored in acetone. The dehydrated samples were subsequently dried under liquid CO2. The samples were mounted on an SEM mounting stub with double-coated tape, sputter-coated with gold [47] and examined via a Zeiss DSM 940 electron microscope at the SEM unit of Assiut University.

2. Diagnosis of arthropods isolated from fly exoskeletons
  Morphological diagnosis

The tiny arthropods isolated from the fly exoskeleton were washed with PBS, centrifuged, and concentrated by ultracentrifugation for 5 min. Lactophenol was used to prepare and clear the mite samples [48]. The samples were subsequently examined via a binocular microscope.

- Molecular diagnosis

Microscopically unidentified external arthropod larvae on the fly exoskeleton were preserved in 70% ethanol for molecular analysis and processed as soon as possible, minimizing DNA degradation. Genomic DNA was extracted via a QIAamp® DNA Mini Kit (QIAGEN, Cat. no. 51304). Conventional PCR was performed with universal primers targeting the mitochondrial *COX1* gene following Folmer et al*.* [49]

### Detection of *Cryptosporidium* infection in diarrheic animals

In September and October 2020, raised animals at site B experienced an acute diarrheal outbreak. Therefore, a cross-sectional study was done to detect the presence of parasitic infections, particularly, *Cryptosporidium.* Fresh stool samples from diarrhea-infected animals were collected in separate sterile containers. The samples were taken to the laboratory at the Faculty of Science, Assiut University, for examination. Approximately five grams of each collected stool sample was subjected to a direct smear and modified with Ziehl–Nielsen stain to detect *Cryptosporidium* infection.

### Sequencing and phylogenetic analysis of *Cryptosporidium* spp. and external arthropod larvae

PCR amplicons were sequenced (Applied Biosystems, Foster City, CA, USA). The obtained DNA sequencer chromatograms were edited and assembled into a consensus sequence via ChromasPro software. Contigs were checked, trimmed, and compared for homology in the GenBank database via BLAST (http://www.ncbi.nlm.nih.gov). Multiple alignment and evolutionary analyses were conducted via MEGA11 software. The evolutionary history was inferred via the neighbor‒joining method, and the evolutionary distances were calculated via the maximum composite likelihood method. The resulting tree was drawn to scale.

### Statistical analysis

All the statistical analyses were performed via IBM SPSS 26.0 software. Categorical variables such as the number of collected flies and parasite prevalence were described as numbers and percentages (N, %). The chi-square and Fisher’s exact tests were used to compare categorical variables. The power analysis was performed using G*Power software to ensure that the sample size was adequate to detect significant differences in fly prevalence and the isolated across seasons (≥ 80% power and a significance level (α) = 0.05). A two-tailed *p*-value < 0.05 was considered statistically significant.

## Results

This study revealed a total of 12,749 flies belonging to seven dipteran families, namely, Muscidae (7,978), Sphaeroceridae (2,910), Fannidae (1,134), Ulidiidae (137), Sepsidae (581), Calliphoridae (7), and Sarcophagidae (2), from the three targeted sites. The highest number of flies were collected from animal rearing site B (5890, 46.20%), followed by site A (4323, 33.91%), and the lowest number of flies were collected from site C (2536, 19.89%). The family Muscidae was the most prevalent family at all three sites (62.58%), followed by the families Sphaeroceridae (22.83%), Fannidae (8.89%), Sepsidae (4.56%) and finally Ulidiidae (1.07%). The families Calliphoridae (0.055%) and Sarcophagidae (0.02%) were the least common families at all collection sites. Additionally, the family Sarcophagidae was not detected at site C during the study. (Table 1).

**Table 1:** Dipteran families and parasite infection distribution across three animal-rearing sites in Assiut Governorate, Upper Egypt

Dipteran family	Fly species	Collection Site	No. of flies	Total No	Total no. of fly pools	Positive pools infected with parasites (n/%)
**Muscidae**	*Musca domestica* Linnaeus, 1758	A	2628	7715	772	746 (96.6%)
		B	3668			
		C	1419			
	*Musca sorbens* Wiedemann, 1830	A	42	65	8	8 (100%)
		B	23			
		C	0			
	*Stomoxys calcitrans* (Linnaeus, 1758)	A	12	198	22	21 (95.6%)
		B	19			
		C	167			
**Sphaeroceridae**	*Borborillus vitripennis* (Meigen, 1830)	A	900	2910	292	238 (81.5%)
		B	1240			
		C	770			
**Fanniidae**	*Fannia canicularis* (Linnaeus, 1761)	A	431	1134	116	111 (95.7%)
		B	621			
		C	82			
**Sepsidae**	*Sepsis punctum* (Fabricius, 1794)	A	178	495	56	52 (92.8%)
		B	277			
		C	40			
	*Meroplius minutus* (Wiedemann, 1830)	A	25	86	15	14 (93.3%)
		B	22			
		C	39			
**Ulidiidae**	*Physiphora alceae* (Preyssler, 1791)	A	105	137	20	20 (100%)
		B	16			
		C	16			
**Calliphoridae**	*Calliphora vicina* Robineau-Desvoidy, 1830	A	0	2	2	2 (100%)
		B	1			
		C	1			
	*Chrysomya megacephala* (Fabricius, 1794)	A	0	2	2	2 (100%)
		B	2			
		C	0			
	*Lucilia sericata* (Meigen, 1826)	A	1	3	3	3 (100%)
		B	1			
		C	1			
**Sarcophagidae**	*Sarcophaga* sp.	A	1	2	2	2 (100%)
		B	0			
		C	1			
**Total**				12,749	1310	1219 (93%)

The results of the present study revealed that the density of fly populations across various species was high in spring and summer, followed by autumn. In contrast, the fly density decreased during the winter**.** The fly species presented in each family were *M. domestica, M. sorbens* and *Stomoxys calcitrans* (Muscidae); *Physiphora alceae* (Ulidiidae); *Meroplius minutus* and *Sepsis punctum* (Sepsidae); *Borborillus vitripennis* (Sphaeroceridae); *Calliphora vicina, Chrysomya megacephala,* and *Lucilia sericata* (Calliphoridae); *Sarcophaga* sp. (Sarcophagidae); *and Fannia canicularis* (Fanniidae) (Additional file 1: Fig. S1).

### Parasites isolated from the collected fly pools

**Fig. 2:**
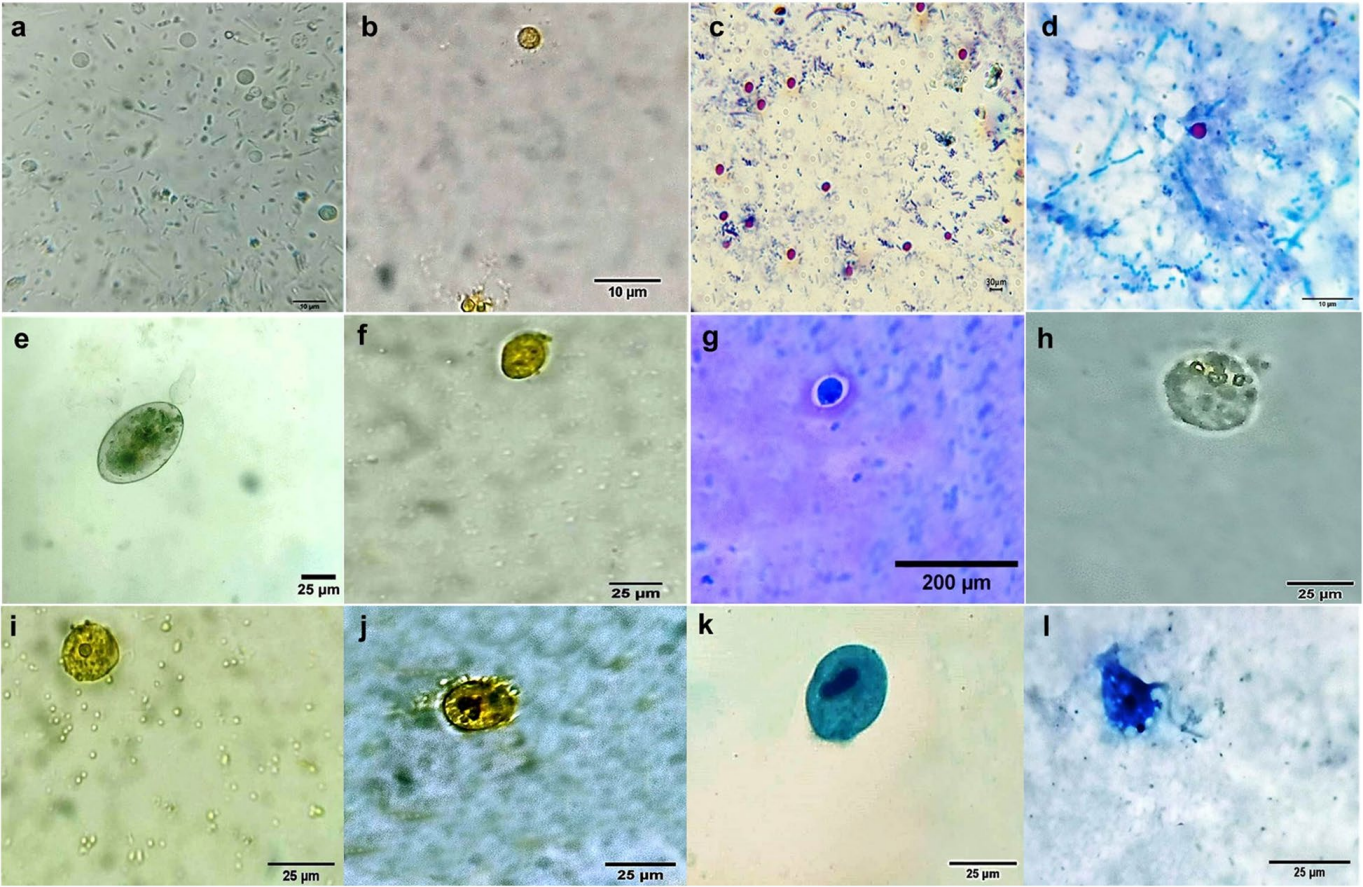
Micrographs show protozoan parasites isolated from fly pools stained with different stains. *Cryptosporidium* spp. oocysts, **a** = Direct smear, **b** = Iodine stained, and **c**, **d** = Ziehl–Nielsen stained, 100 × oil immersion. *Giardia* spp. cyst, **e** = Direct smear, **f** = Iodine stained, and **g** = Methylene blue stained 40x. *Entamoeba* spp. cyst, **h** = Direct smear, **i** = Iodine stained 40x. *Balantidium* coli trophozoites, **j** = iodine-stained, **k** = methylene blue-stained 40x. l = *Trichomonas* spp. trophozoites stained with methylene blue, 40x

**Fig. 3:**
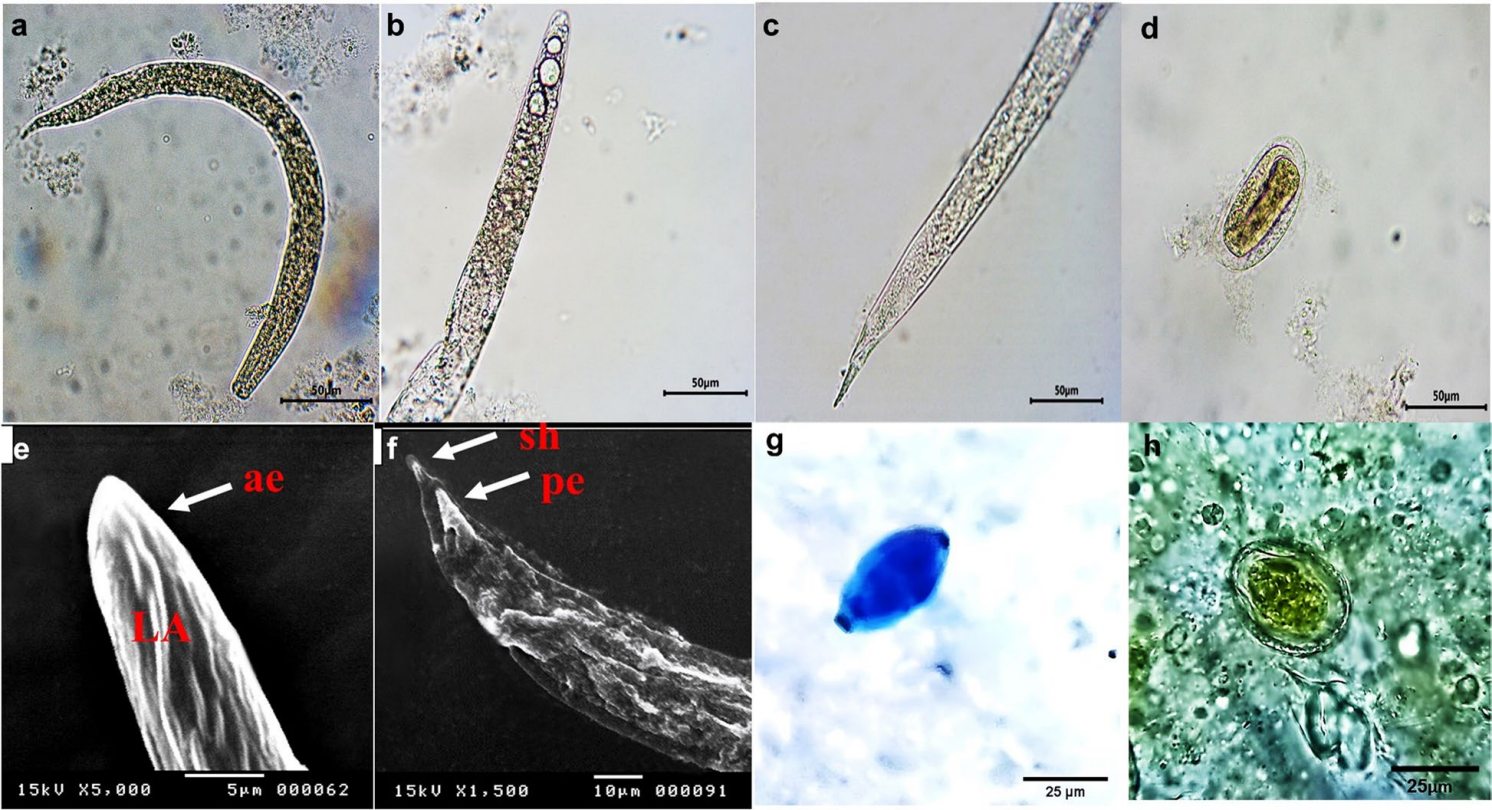
The micrographs show the helminth eggs and larvae isolated from the fly pools. **a**-**h** *Trichostrongylids* whole 1st stage larva (**a**), larval anterior end showing numerous intestinal cells (**b**), posterior end of first stage larvae showing short, pointed sheath (**c**), and *Trichostrongylids* egg (**d**). A scanning electron micrograph of *Trichlostrongylids* larvae showing lateral alae (LA) at the anterior end while the esophagus is not well demarcated (**e**), posterior end (pe) showing a pointed tail and short sheath (sh) (**f**). **g**: *Trichuris* spp. immature egg, (**h**): *Toxocara vitulorum* egg. Lens 40 × and 10x

Microscopic inspection of the collected parasites revealed the presence of *Cryptosporidium* spp*., Entamoeba* spp*., Balantidium* spp*.,* and *Giardia* spp. cysts. Additionally, we identified a sporadic infection with *Trichomonas* parasites (Fig. 2). Furthermore, we detected parasitic helminths, such as *Trichostrongylidae* eggs and larvae, as well as *Trichuris* spp. eggs on the flies. We recorded a sporadic infection with *Toxocara vitulorum* eggs during the study (Fig. 3).

**Fig. 4:**
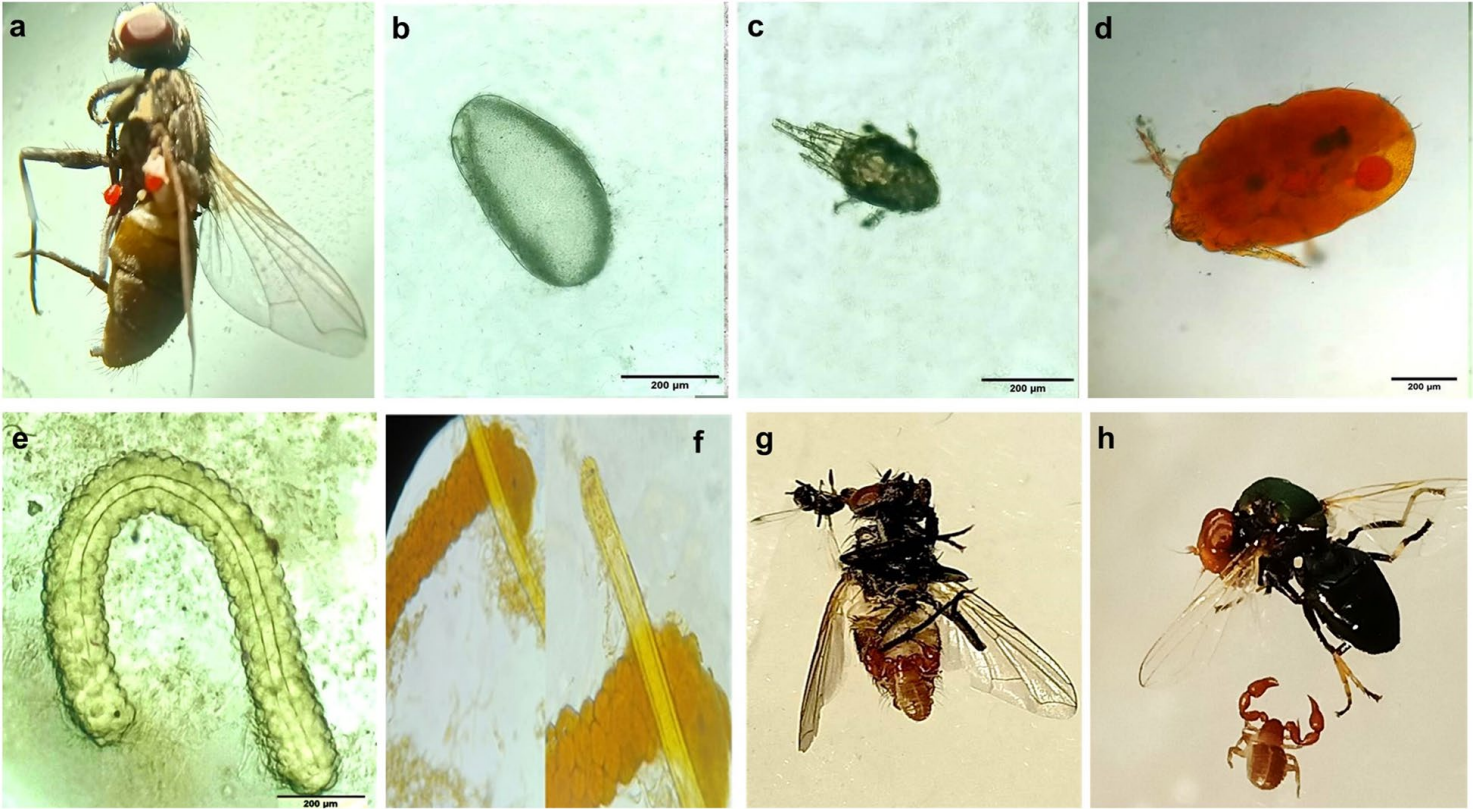
External arthropods detected on fly exoskeletons showing different stages of mites (eggs, nymphs, and adults) (**a**-**d**). **e** & **f**: Midge larvae were detected via direct smearing, iodine-stained and coinfested with other nematode larvae. **g** & **h**: Pseudoscorpions infesting *Musca domestica* and *Physiphora alceae* fly exoskeletons, respectively

Our examination also revealed the presence of tiny arthropods, including mite eggs, nymphs, and adults, on the skeleton of the studied flies. Sporadic infections with tiny, oval-shaped scorpion-like arachnids ending with two pincers called pseudoscorpions were recorded attached to the exoskeletons of *M. domestica* and *Physiphora alceae* at site B. In addition, the present study revealed high infestation levels of the fly exoskeleton by midge larvae only in flies collected from site B. The larvae were slender, long larvae called bloodworms due to the presence of a hemoglobin analog, making them look red (Fig. 4).

### Diversity and seasonal abundance of the detected parasites

- Detection of protozoan parasites

The study found that *Cryptosporidium* was the most common parasite in flies, with infection rates between 64.4% and 100% across species. Microscopy revealed various species carried *Cryptosporidium* in all seasons, with lower intensity in winter and peak rates of 100% in *Fannia canicularis* during autumn. Notable infected species included *M. domestica* (81–98.9%), *Borborillus vitripennis* (64.4–86.7%), and *Fannia canicularis* (89.5–100%).

*Entamoeba* was mainly found in *M. domestica* during summer and spring, with prevalence from 22.6% to 90.1%, while *Fannia canicularis* had rates from 10.5% to 74.4%, and *Borborillus vitripennis* ranged from 11.1% to 50%. Some species, like *Musca sorbens* and *Meroplius minutus*, even reached up to 100% infestation.

*Balantidium* infection also showed high prevalence, particularly at site B during summer and spring. *M. domestica* and *Sepsis punctum* had infection rates from 8.9% to 100%. *Fannia canicularis* and *Physiphora alceae* ranged from 44.2% to 88.2% and 56.6% to 94.1%, respectively. *Borborillus vitripennis* varied from 37.2% to 91.4%. *Musca sorbens* and *Stomoxys calcitrans,* despite lower sample sizes, had infection rates of 50% and 33.3% to 83.3%, respectively.

*Giardia* infection varies among fly species, with overall prevalence lower than other parasites. *Musca sorbens* and *Stomoxys calcitrans* had the highest infection rates, ranging from 16.7% to 50% and 12.5% to 20%, respectively, despite being less abundant. Moderate rates were found in *Fannia canicularis* (2.56% to 20%), *Sepsis punctum* (5.26% to 6.25%), and *Physiphora alceae (*16.67% to 33.3%). *M. domestica* and *Borborillus vitripennis* had the lowest rates (0.8% to 1.3% and 2.04% to 3.64%). Despite *Giardia* is less prevalent but identified in various fly species, particularly in summer and spring months, mainly at site B (Additional file 2: Tables S1–S8).

- Detection of helminth parasites

*Trichuris* infection varied among the studied fly species. *Musca domestica* was contaminated with *Trichuris* eggs, with a prevalence ranging from 3.7– 41%. *The prevalence rates of sepsis punctum*, *Borborillus vitripennis*, and *Fannia canicularis* were lower, ranging from 12.5–20%, 4.4–18.18%, and 8.8–13%, respectively. *Musca sorbens*, *Stomoxys calcitrans*, and *Physiphora alceae* presented minimal to no *Trichuris* infection.

The results indicated a significant presence of Trichostrongylidae eggs and larvae, particularly in autumn and spring. *Musca sorbens* showed a high prevalence rate of 41.67%, peaking at 100% in autumn similar to *sepsis punctum*. Other species, including *M. domestica, Borborillus vitripennis*, and *Fannia canicularis,* had lower infection rates of 20% to 66.67%. Nematode infections were found only at sites A and B, and Trichostrongylidae larvae were carried by all reported fly species except *Chrysomya megacephala* and *Sarcophaga sp*. (Additional file 2: Tables S1– S8).

- Detection of arthropod ectoparasites on fly exoskeletons

The exoskeleton of the fly showed high mite infestation rates year-round, particularly in *M. domestica* during summer and autumn (77–100%). Autumn was peak season for *Physiphora alceae* and *M. sorbens,* with infestations ranging from 66.7% to 100%. *Stomoxys calcitrans* displayed fluctuations between 12.5% and 100% infestation, while *Fannia canicularis* and *Meroplius minutus* had rates from 26 to 100%, predominantly in autumn.

Surprisingly, the mite eggs and larvae were found on the exoskeletons of the collected flies, mainly from sites A and B. All the fly species detected in the study, except *Borborillus vitripennis* and *Sepsis punctum*, carried different mite stages. (Additional file 2: Tables S1–S8).

The study found significant midge infestations in flies from site B, with 595 fly pools examined. Of these, 310 pools (52.1%) were infested. Notable infestation rates were seen in *M. domestica* (42.5%), *Borborillus vitripennis* (16.5%), and *Fannia canicularis* (8.8%). Additionally, species like *Musca sorbens, Meroplius minutus,* and *Calliphora vicina* had 100% infestation rates, though fewer ample sizes (Additional file 2: Table S9).

In this study, fly species from the families Calliphoridae and Sarcophagidae were found in small numbers across 2–3 pools. *Calliphora vicina* was collected at site B in autumn (heavily infested with protozoa and helminths) and spring (with a less diverse parasite load). *Chrysomya megacephala* was found at site B in summer and spring, with the summer sample carrying *Cryptosporidium* spp., *Entamoeba* spp., *Balantidium* spp. and *Trichuris*. *Lucilia sericata* was collected from sites B and C in summer and site A in spring, showing multiple parasites in summer and oocysts/cysts in spring. *Sarcophaga* sp. was collected from sites A and C in summer and spring, respectively, also infested with *Cryptosporidium* spp*., Entamoeba* and mites.

### Detection of *Cryptosporidium* infection in diarrheic animals

In September and October 2020, 171 animals at site B experienced an acute diarrhea outbreak. Stool examinations confirmed *Cryptosporidium* infection in 45.6% of the animals. The infection was significantly more prevalent in young calves and growing animals (< 1 year) *(P-* value = 0.001), as shown in Table 2.

**Table 2:** Prevalence of *Cryptosporidium* infection in diarrheic animals during the outbreak in September and October 2020 at animal site B

Animal age groups	Infected (*n* = 78)	Free (*n* = 93)	Total (*n* = 171)
Baby calves (1 day-1 month)	18 (100%)	0 (0%)	18
Preweaned calves (1–3 months)	18 (90%)	2 (10%)	20
Weaned (3–7 months)	16 (80%)	4 (20%)	20
Growing (7–12 months)	26 (70.3%)	11 (27.7%)	37
Elders (1–2 years)	0 (0%)	27 (100%)	27
Elders (2–3 years)	0 (0%)	25 (100%)	25
Elders (3–5 years)	0 (0%)	24 (100%)	24

### Molecular diagnosis and phylogenetic analysis of *Cryptosporidium*

**Fig. 5:**
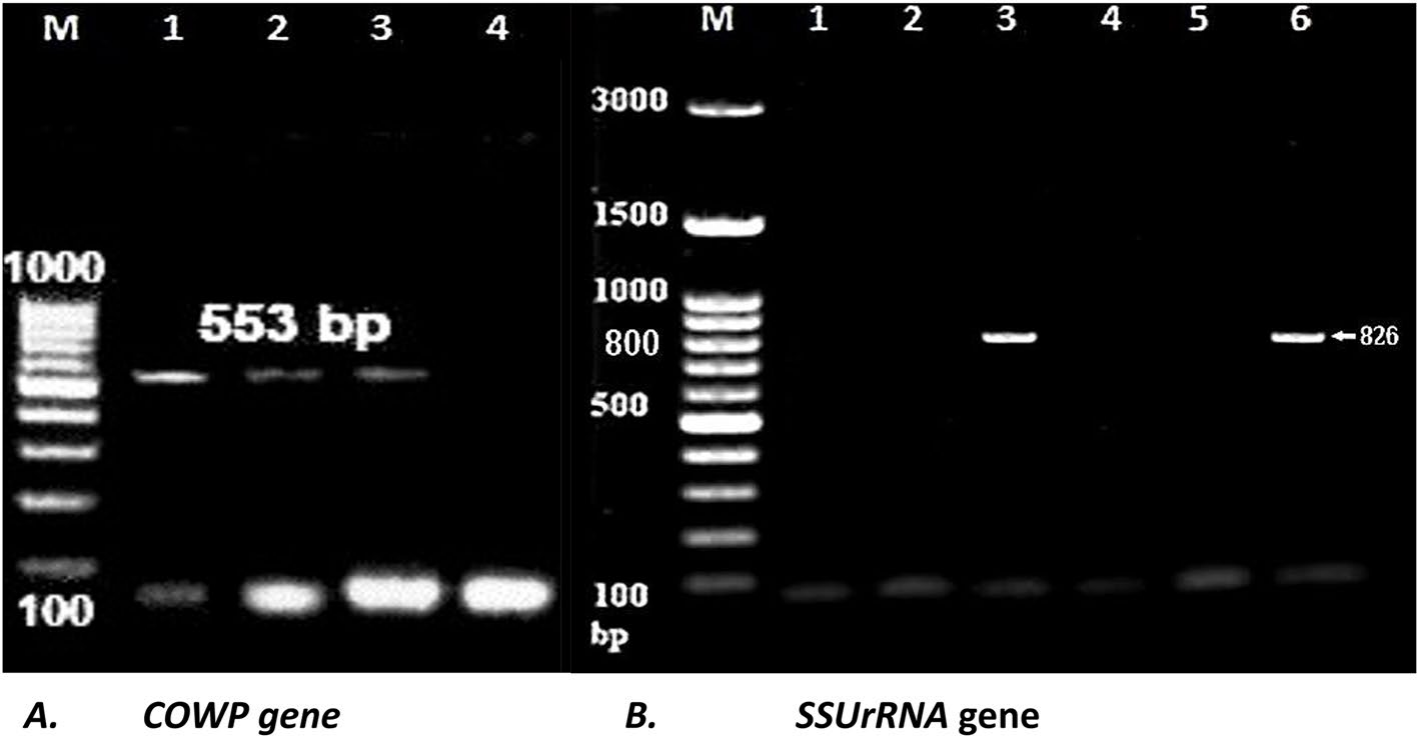
Nested PCR amplification of the *Cryptosporidium COWP* gene (**A**) and the *SSUrRNA* gene (**B**) resolved on a 1.5% agarose gel stained with ethidium bromide. Positive PCR products at 553 bp are shown. for the COWP gene and positive amplification products of the SSUrRNA gene in lanes 3 and 6, with single bands at 826 bp. Lane (1A): positive control, lanes (4A and 1B): negative control. (M): DNA marker (Thermo Fisher Scientific, Cat. no. SM0243)

Microscopically positive fly washes for *Cryptosporidium* were further confirmed by nested PCR targeting the *COWP* gene, which yielded a positive amplification product at 553 bp. In comparison, the *SSUrRNA* gene produced a positive amplification at 826 bp (Fig. 5).

**Fig. 6:**
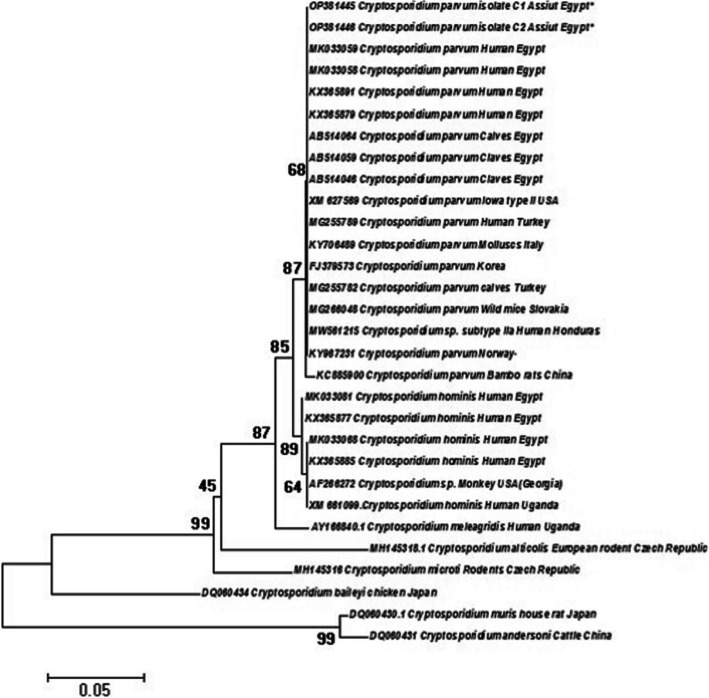
Phylogenetic tree of *parvu*m cysts inferred via neighbor‒joining analysis of the *COWP* gene sequence. *Cryptosporidium muris* (house rat) and *Cryptosporidium andersoni* (cattle) from China were used as the out-group. Nonparametric bootstrap values (based on 1000 replicates) are shown at each node. The scale bar indicates the number of substitutions per site

The sequences generated from the *Cryptosporidium* amplified amplicons were deposited in the GenBank database under accession numbers OP381445 and OP381446 for the *Cryptosporidium COWP* gene (Fig. 6), and the retrieved sequences were found to belong to the zoonotic species *Cryptosporidium parvum*. It showed high similarity with isolates from various locations worldwide, including Egyptian isolates and isolates from Turkey, Slovakia, Norway, and China. They also exhibited close similarities with *C. parvum* subtype II of the IOWA isolate from the USA (XM627569) and subtype II of the human isolate (MW561215) from Honduras, with a high bootstrap confidence of 87%. The species most closely related to our isolate was *C. hominis* in humans from Egypt, with 89% homology.

### Midge genotyping and phylogenetic analysis

**Fig. 7:**
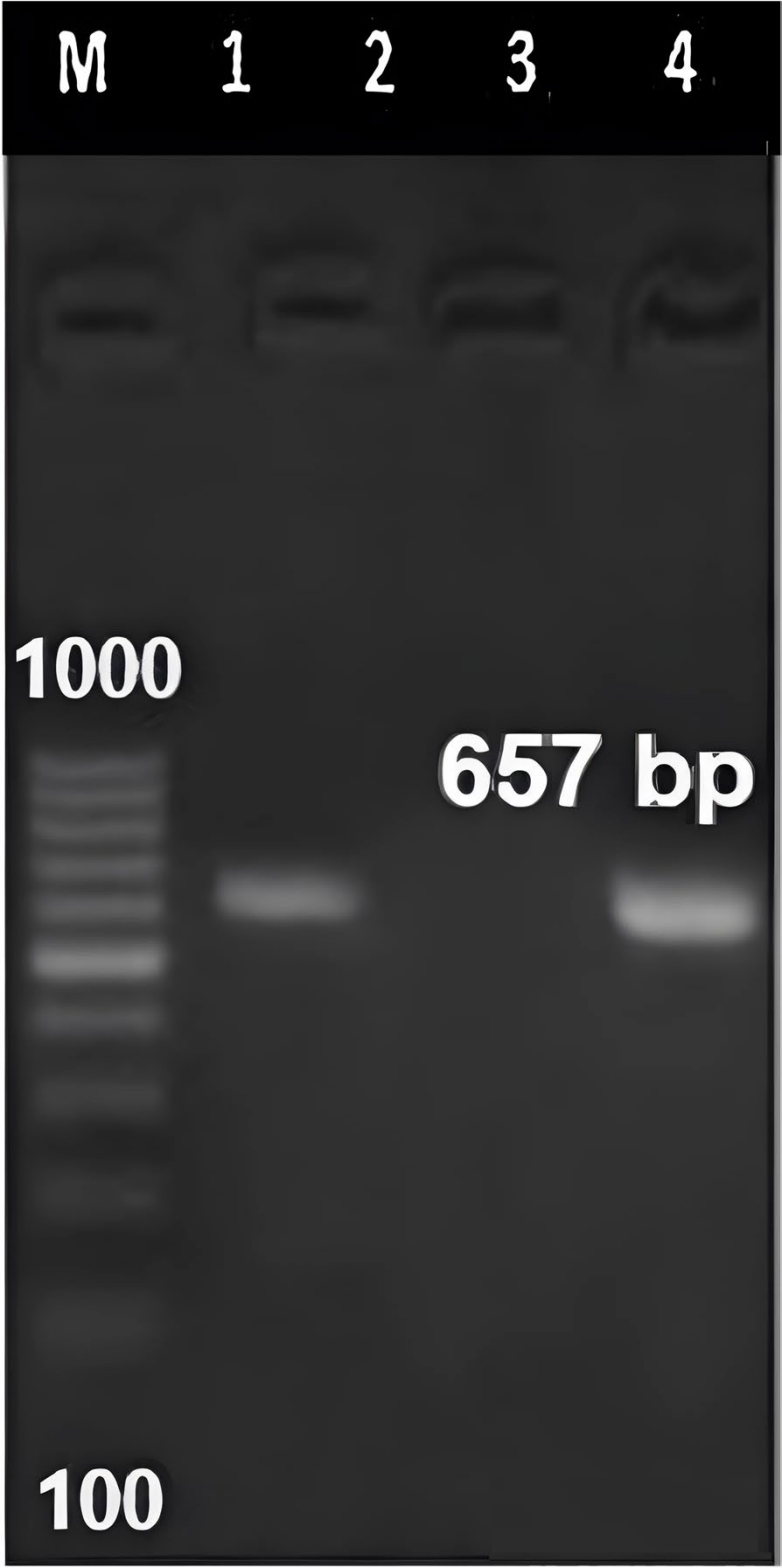
DNA amplification of the *COX1* gene in chironomid larvae isolated from fly washes. The PCR products were resolved via 1.5% agarose gel electrophoresis and stained with ethidium bromide. The gel image shows positive PCR products in lanes (1, 4), with single bands at 657 bp. Lane (1): positive control, lane (2): negative control. (M): DNA marker (Thermo Fisher Scientific, Cat. no. SM0243)

**Fig. 8:**
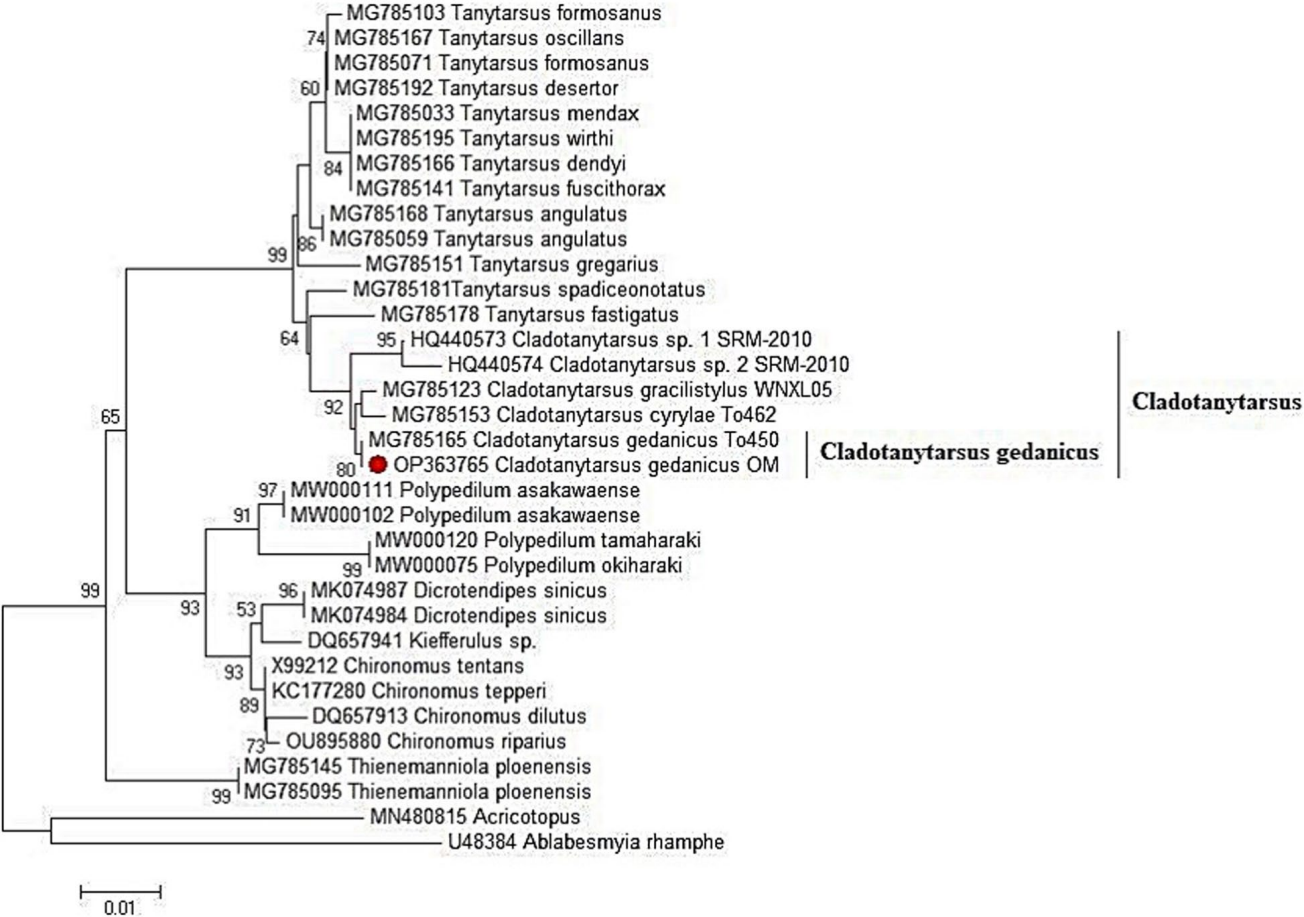
Phylogenetic tree of chironomid larvae inferred via neighbor-joining analysis of the *COX1* gene sequence of the Chironomidae family. The red dots represent the sequences obtained in this study. *Acricotopus* and *Ablabesmyia rhamphe* were used as the out-group. Nonparametric bootstrap values (based on 1000 replicates) are shown at nodes. The scale bar indicates the number of substitutions per site

Sequence analysis of the PCR amplification-derived 657 bp amplicon (*COX1* gene) (Fig. 7) revealed identity with the *Cladotanytarsus gedanicus COX1* gene (Poland, accession number MG785165), which was deposited in GenBank under accession number OP363765. Blast analysis revealed that the retrieved sequences belonged to *Cladotanytarsus gedanicus*, a species belonging to the Chironomidae family. We used the Muscle algorithm to compare our isolates with GenBank sequences, constructing a phylogenetic tree based on *COX1* gene sequences. Our isolate was identical to *Cladotanytarsus gedanicus* from Poland and highly similar to other *Cladotanytarsus* species from different countries. It also shares high similarity with *Tanytarsus* species, with a homology of up to 98.5%. Both species were grouped into a separate subtree with a 99% bootstrap value (Fig. 8).

## Discussion

The present study was conducted at different animal-rearing sites in the Assiut governorate, Upper Egypt, where animal livestock, including cattle and buffaloes, are raised. This study focused on animal farms close to human households and explored potential contact between flies and reared animals, which poses a risk to nearby humans. Animal farms often present a greater population of filth flies [28]. Our findings revealed a high abundance of filth flies at the studied sites, which could be attributed to the warm climate in Upper Egypt, which accelerates their reproduction and development [50]. Moreover, poor sanitation practices significantly impact the potential increase in synanthropic flies in animal and human habitations due to the availability of food and breeding places [51]. Furthermore, the presence of several agricultural activities in the bordering regions of the study sites, as these animal farms are located primarily in rural areas, could imply the widespread use of pesticides and fertilizers in these locations, which affects the natural killers of filth flies, increasing fly density [52].

The number of collected flies varied from one site to another. Compared with the other sites, animal site B had the greatest number of flies collected, indicating its dominance in the fly population. This may be attributed to poor periodic sanitation at the animal farm at site B and its proximity to human households, which could increase fly contact with humans and subsequently lead to increased fly abundance [24]. Additionally, most animals raised at site B experienced acute episodes of diarrhea, so more flies were attracted to this site.

The findings of the present study revealed that the warm season had a high fly density, which coincided with the findings of a previous report from Sohag, Upper Egypt, which indicated that spring and summer were the most conducive seasons to fly activity across various species, followed by autumn, whereas the number of flies decreased during the winter [53]. This finding suggests that the differences in fly numbers across seasons are influenced primarily by climate factors, particularly temperature [54]. The influence of environmental factors and seasonal distribution on fly abundance in the studied sites has been extensively investigated in a prior study conducted by our research group [55]. Additionally, the present results are consistent with the findings of Eesa and el-Sibae, who reported that synanthropic fly species were most abundant in May and least common in January [50].

The present results revealed that *M. domestica* was the most prevalent and abundant fly species, accounting for more than 60% of all the collected flies at the three collection sites and throughout the study period. This dominance is consistent with previous studies conducted in Upper and Lower Egypt and globally [56, 54]. The warm climate of Upper Egypt, coupled with abundant organic waste, particularly animal manure, provides ideal breeding conditions for this species. Additionally, their ability to thrive in both rural and urban environments, close association with human activities and diverse feeding habits further contribute to their widespread presence [2, 57, 58]. These species reportedly carry numerous parasites and pathogens that can cause significant health hazards in humans and animals. Numerous studies in Iraq and Sudan have shown that houseflies can transmit parasites such as *Cryptosporidium, Cyclospora, and Entamoeba,* as well as different helminth eggs and larvae, leading to outbreaks in humans and animals [59, 60]*.*

In this study, filth flies were carriers of many protozoan cysts, such as *Cryptosporidium, Giardia, Balantidium, Entamoeba,* and even *Trichomonas* trophozoites, as well as nematode eggs and larvae, such as *Trichuris, Trichostronglidae,* and *Toxocara*. These findings align with previous studies that have shown that various species of synanthropic flies carry numerous protozoan cysts, as well as eggs of helminths such as *Ascaris lumbricoides, Trichuris trichiura, Enterobius vermicularis, Capillaria hepatica, Taenia* sp.*, Hymenolepis nana, Toxocara canis,* hookworm*,* and *Strongyloides stercoralis* larvae [61]

The findings of this study revealed that *Cryptosporidium* spp. oocysts are highly prevalent among the collected flies. Across the three animal-rearing sites, the incidence rate of this parasite ranged from 64 to 100%, indicating its widespread presence among multiple fly species. The high prevalence of *Cryptosporidium* in the collected fly species may be attributed to the sensitive detection methods used in this study, which depend on the pooling of fly samples, the use of a flotation concentration technique to enhance the detection of *Cryptosporidium* oocysts and the use of specific Ziehl–Nelsen stains [24]. Additionally, certain fly species were present in a few numbers, impacting the statistical reporting of samples. Moreover, infected animals at these collection sites can contribute to parasite transmission to flies. This finding was supported by a cross-sectional study at animal farm site B, where an acute diarrheal outbreak occurred in September and October 2020. This study confirmed the coexistence of *Cryptosporidium* infection in animals experiencing acute diarrhea, with a high infection rate of up to 45.6%. Young calves and growing animals (less than 1 year old) were more affected than older animals were. These data are consistent with findings from other studies regionally and worldwide [62, 63]. The higher prevalence among younger animals may be due to their lower body immunity, making them more susceptible to infection. However other factors contributing to diarrhea in animals on this site cannot be entirely ruled out and need further investigations.

The proximity of cattle and other domestic animals to human households, particularly in rural areas, poses a significant risk of zoonotic diseases. Cattle manure is a prime breeding ground for filth flies and a source of *C. parvum* oocysts, which are a prevalent cause of diarrhea in Egypt with confirmed zoonotic potential [29, 64]. Previous studies have shown that flies near cattle farms and landfills can transmit *Cryptosporidium* and *Giardia* from unsanitary sources, potentially leading to cryptosporidiosis and giardiasis in humans and animals [34, 53, 61].

Cryptosporidiosis is considered a significant health problem in Assiut Governorate, Upper Egypt, with high infection rates in animals, particularly diarrheic cattle calves (45.16%) and buffalo calves (33.96%) [65]. Samples collected from a site where animals had diarrhea, suggest a sporadic outbreak of *Cryptosporidium* may not reflect the general prevalence of this parasite in flies across the region. Parasite prevalence might be more accurate with broader sampling and spatial variations. Furthermore, water sources at Assiut University Hospital were found to be contaminated, with 79% of samples testing positive for *Cryptosporidium* oocysts and higher contamination levels in summer [66]. On the other hand, the number of recorded human infections in Assiut is lower, at 3.3% in children and 12% in ICU patients [67, 68]. The higher prevalence of *Cryptosporidium* infection in flies compared to other parasites might be due to the use of more sensitive molecular detection differences in diagnostic sensitivity across methods.

In Egypt, *E. histolytica* infection has a wide prevalence ranging from 0–57% in diarrheic patients, mainly in poor districts and Upper Egypt [69]. In the present work, *Entamoeba* was carried by all species of the studied flies with high prevalence, mainly in summer, spring, and autumn, at the three collection sites, especially at site B. The role of filth flies as carriers for *Entamoeba* spp. was previously reported in other studies worldwide [70, 71]. Moreover, most of the collected flies carried *Balantidium* trophozoites and cysts throughout all seasons, with high infection rates in warm seasons, especially at site B.

*Giardia lamblia* also represents a predominantly common intestinal parasite in Assiut governorate, Egypt, particularly among children, with infection rates of up to 24.2% [72]. Flies are essential carriers of *Giardia* cysts. In our study, *Giardia*, although less prevalent than other protozoans, was still identified in various fly species, particularly during the summer and spring months, mainly at site B. This finding is consistent with previous studies in dairy farms in China and two towns in Aragón, Spain, where fly batches tested positive for *Giardia* infection [73, 24].

The study revealed that several fly species were infected with nematode parasites, particularly *Trichuris* and *Trichostrongylidae*; however, they presented low to moderate infection rates. *Musca domestica* was the species most commonly infected with *Trichuris*, while *Musca sorbens* and *Sepsis punctum* had the highest prevalence of *Trichostrongylidae,* particularly in autumn from sites A and B.

The low to moderate rates of nematode contamination may be attributed to several factors, including seasonal variations and environmental conditions that affect the developmental stages of these parasites [74]. The viscous surface of some nematode eggs enhances their attachment to the flies' bodies [32]. Additionally, poor hygiene practices on these animal farms may contribute to the long persistence of these parasitic stages [74]. Specifically, nematode species that do not require intermediate hosts (direct life cycle) and are transmitted through soil pose an increased risk of transmission via flies [75, ]. These findings are in agreement with those of previous studies [71, 76]. Additionally, a study conducted by Förster and colleagues indicated that houseflies (*M. domestica*) can act as mechanical vectors for various intestinal parasites, including *Ascaris suum, Strongyloides ransomi, Metastrongylus* spp.*, and Trichuris suis* [32].

This study in Assiut Governorate, Upper Egypt, is the first to record flies infested with external arthropods such as mites, midge, and pseudoscorpions. The flies collected at site B were significant carriers of midge larvae, with a prevalence of 52.1%. This highlights the widespread occurrence of midge infestations, particularly among families Muscidae, Sphaeroceridae, Fanniidae, and Sepsidae. These insects are known to cause economic burdens, nuisances, and health problems, including allergic reactions [77]. Previous research has not explored the relationship between chironomid larvae and filth flies. However, the significant presence of midge larvae in fly samples from site B may be due to water contamination from nearby animals. A previous study found that cattle grazing activities, such as disturbing pond margins and defecating near ponds, likely contribute to water turbidity. This, in turn, has a negative impact on the water quality of farm ponds, leading to the predominance of chironomid species such as *Chironomus* and *Glyptotendipes*.[78].

Additionally, the examined flies carried various stages of mites that match previous reports that have shown that mites utilize a variety of insects, such as beetles, synanthropic flies, and dragonflies, for transportation (phoresy) and as a source of food (parasitism), where they feed on their hemolymph. When insects come into contact with animals, they can become infected with mites and transmit them to other locations or hosts [79].

Importantly, many of the synanthropic fly species collected in the present study were found to carry multiple parasites simultaneously, which is known as coinfestation. This highlights the significant health risk posed by synanthropic flies. A study by Liu et al. demonstrated that nonbiting flies could carry more than 100 pathogens [80].

In summary, the findings of this study indicate that seasonal fluctuations have a substantial impact on parasite prevalence in flies, with *Cryptosporidium* being the parasite most frequently encountered, reaching levels of 64.4–100% in warmer months. In addition to *Entamoeba* and *Balantidium*, a higher prevalence of these parasites is also observed in the summer months, specifically in *M. domestica* and *Sepsis punctum*. *Giardia* is less prevalent but more frequent in spring and summer, whereas helminths such as *Trichuris* and *Trichostrongylidae* reach their peak in autumn and spring, particularly in *Musca sorbens.* Additionally, ectoparasites such as mites and midges become more abundant during warmer months. These findings highlight the need for targeted fly control during these seasons to reduce parasite transmission. Furthermore, conducting a multivariate analysis of the factors influencing fly distribution and parasite density including environmental factors could provide valuable insights into the ecological determinants of fly populations and parasite abundance.

The present study used molecular techniques such as PCR and sequence analysis to enhance the identification and characterization of parasites. Specifically, this study targeted the *COWP* and *SSUrRNA* genes via a nested PCR assay to confirm *Cryptosporidium* infection. Phylogenetic analysis of the *Cryptosporidium* sequences revealed that they belong to the zoonotic species *C. parvum*. The sequences of *C. parvum* subtype II of the IOWA isolate from the USA and subtype IIa from a human isolate in Honduras were highly similar to our isolates. This finding indicates that the collected flies play a role in transmitting *Cryptosporidium* to animals at the studied sites and may pose a potential risk to humans. Furthermore, genomic analysis was performed to identify the midge species isolated from the fly pools that target the mitochondrial *COXI* gene, and the retrieved sequences were found to belong to *Cladotanytarsus gedanicus*, which are chironomids. The survey of only chironomid midges conducted in Egypt resulted in the capture of species of *Cladotanytarsus kieffer* [84]. This is the first report identifying these midge species in Egypt.

### Study limitations

This study provides valuable insights, but it has limitations because its focus is on a specific region in Upper Egypt, which affects the generalizability of the findings. The parasites detected on the synanthropic filth were primarily identified via microscopic observations of eggs and cysts via concentration techniques and staining. Although this method is widely used and considered the gold standard for detecting certain parasites, such as *Cryptosporidium*, it may overestimate prevalence rates. Therefore, molecular identification offers strong specificity and high sensitivity. However, this approach requires expensive and sophisticated equipment and specialized personnel. So, future studies could combine microscopy with sensitive molecular techniques, such as PCR and qPCR, for better detection. Developing cost-effective diagnostic tools will improve early detection in resource-limited areas while expanding sampling to target different sites other than animal farms will help identify high-risk areas for infection. In addition, the lack of human samples to determine the prevalence of these parasites in the local human population could influence the assessment of the actual risk associated with the potential of these flies to carry zoonotic parasites. Further research is required on multivariate analyses and into the prevalence and genetic diversity of *Cryptosporidium* and other zoonotic parasites among farm workers and people living nearby to gain a better understanding of transmission risks, assess public health risks, and inform targeted interventions.

## Conclusion

The present study highlights the most prevalent synanthropic filth fly families and species distributed in three animal-rearing sites in Assiut Governorate, Upper Egypt. Muscidae, especially *Musca domestica,* had the highest prevalence of all the collected flies and carried the greatest number of parasites. *Cryptosporidium* spp. was the most prevalent parasite carried by the collected flies, linked to diarrheal episodes in animals, underscoring flies' role as potential zoonotic disease vectors. Seasonal fly prevalence patterns provide valuable insights for optimizing control measures.

To reduce fly abundance and pathogen transmission, we suggest maintaining regular manure removal and enforcing strict sanitary measures while implementing fly control programmes. Future research should focus on investigating the role of flies in transmitting parasites to humans, specifically in environments like markets, restaurants, and waste disposal sites, to better understand the potential of flies as vectors of zoonotic parasites.

## Supplementary Information

Additional file 1. Fig. S1. Photograph showing the fly species collected from the three animal rearing sites in Assiut Governorate, Upper Egypt: (a-c) families Muscidae (*M. domestica*, *M. sorbens* and *Stomoxys calcitrans*, respectively. (d): Family Ulidiidae (*Physiphora alceae*). (e, f) Family Sepsidae (e): *Meroplius minutus*, (f): *Sepsis punctum*). g: Family Sphaeroceridae (*Borborillus vitripennis*). (h-j) Family Calliphoridae (h: *Calliphora vicina*, i: *Chrysomya megacephala*, j:*Lucilia sericata*). (k) Family Sarcophagidae (*Sarcophaga sp*.), family Fanniidae (*Fannia canicularis*).

Additional file 2. Table S1: Seasonal distribution of parasite infestations of *Musca domestica *on the basis of the sedimentation-floatation technique and microscopic examination. Table S2: Seasonal distribution of parasite infestation of *Musca sorbens *on the basis of the sedimentation–floatation technique and microscopic examination. Table S3: Seasonal distribution of parasite infestation of *Stomoxys calcitrans* on the basis of the sedimentation-floatation technique and microscopic examination. Table S4: Seasonal distribution of parasite infestations of *Borborillus vitripennis* on the basis of the sedimentation–floatation technique and microscopic examination. Table S5: Seasonal distribution of parasite infestation of *Fannia canicularis* on the basis of the sedimentation-floatation technique and microscopic examination. Table S6: Seasonal distribution of parasite infestations of *Sepsis punctum* based on the sedimentation-floatation technique and microscopic examination. Table S7: Seasonal distribution of parasite infestations of *M. minutus* on the basis of the sedimentation–floatation technique and microscopic examination. Table S8: Seasonal distribution of parasite infestations of *Physiphora alceae* based on the sedimentation-floatation technique and microscopic examination. Table S9: Seasonal distribution of midge infestations of different fly species collected from site B on the basis of the sedimentation-floatation technique and microscopic examination.

## Data Availability

Sequence data that support the findings of this study have been deposited in the GenBank® database repository (https://www.ncbi.nlm.nih.gov/genbank/), under accession numbers OP381445 and OP381446 for the Cryptosporidium COWP gene and accession number OP363765 for Cladotanytarsus gedanicus COX1 gene.
