# Supplementary figures and images for: Evaluating the role of synanthropic filth flies in the transmission of zoonotic parasites: field and laboratory evidence from different animal rearing sites in upper Egypt with focus on Cryptosporidium spp
Source: BMC Vet Res. 2025 Mar 20;21:188. doi: 10.1186/s12917-025-04627-w (PMC11924607; doi:10.1186/s12917-025-04627-w)

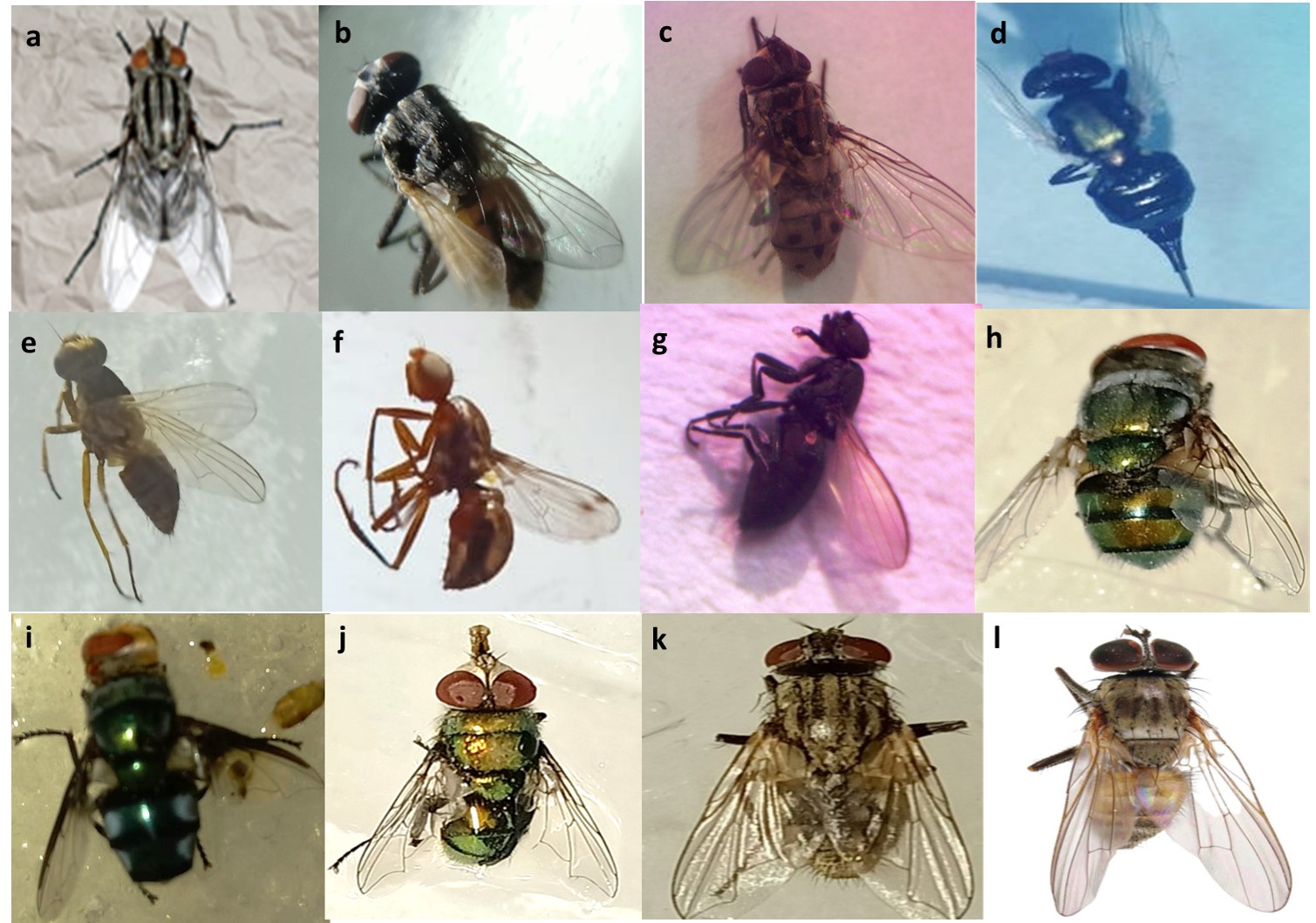

Supplement: Supplementary file 1 — Additional file 1. Fig. S1. Photograph showing the fly species collected from the three animal rearing sites in Assiut Governorate, Upper Egypt: (a-c) families Muscidae (M. domestica, M. sorbens and Stomoxys calcitrans, respectively. (d): Family Ulidiidae (Physiphora alceae). (e, f) Family Sepsidae (e): Meroplius minutus, (f): Sepsis punctum). g: Family Sphaeroceridae (Borborillus vitripennis). (h-j) Family Calliphoridae (h: Calliphora vicina, i: Chrysomya megacephala, j:Lucilia sericata). (k) Family Sarcophagidae (Sarcophaga sp.), family Fanniidae (Fannia canicularis). [file 12917_2025_4627_MOESM1_ESM.tif]
