# Supplementary material for: Evaluating the role of synanthropic filth flies in the transmission of zoonotic parasites: field and laboratory evidence from different animal rearing sites in upper Egypt with focus on Cryptosporidium spp
Source: BMC Vet Res. 2025 Mar 20;21:188. doi: 10.1186/s12917-025-04627-w (PMC11924607; doi:10.1186/s12917-025-04627-w)
Supplement: Supplementary file 2 — Additional file 2. Table S1: Seasonal distribution of parasite infestations of Musca domestica on the basis of the sedimentation-floatation technique and microscopic examination. Table S2: Seasonal distribution of parasite infestation of Musca sorbens on the basis of the sedimentation–floatation technique and microscopic examination. Table S3: Seasonal distribution of parasite infestation of Stomoxys calcitrans on the basis of the sedimentation-floatation technique and microscopic examination. Table S4: Seasonal distribution of parasite infestations of Borborillus vitripennis on the basis of the sedimentation–floatation technique and microscopic examination. Table S5: Seasonal distribution of parasite infestation of Fannia canicularis on the basis of the sedimentation-floatation technique and microscopic examination. Table S6: Seasonal distribution of parasite infestations of Sepsis punctum based on the sedimentation-floatation technique and microscopic examination. Table S7: Seasonal distribution of parasite infestations of M. minutus on the basis of the sedimentation–floatation technique and microscopic examination. Table S8: Seasonal distribution of parasite infestations of Physiphora alceae based on the sedimentation-floatation technique and microscopic examination. Table S9: Seasonal distribution of midge infestations of different fly species collected from site B on the basis of the sedimentation-floatation technique and microscopic examination. [file 12917_2025_4627_MOESM2_ESM.docx]

**Table S1: Seasonal distribution of parasite infestation of *Musca domestica* based on sedimentation-floatation technique and microscopic examination.**

|  | **Positive pools/ Total (%)** | **Site A** | **Site B** | **Site C** | ***P. value*** |
| --- | --- | --- | --- | --- | --- |
| **Summer** | | | | | |
| ***Cryptosporidium*** | **231/243(95.06%)** | 71  (30.7%) | 119  (51.7%) | 41  (17.6%) | <0.001** |
| ***Giardia*** | **2/243(0.8%)** | 0  (0%) | 2  (100%) | 0  (0%) | - |
| ***Entamoeba*** | **219/243(90.1%)** | 51  (23.3%) | 119  (54.5%) | 49  (22.2%) | <0.001** |
| ***Balantidium*** | **154/243(63.37%)** | 26  (16.7%) | 98  (63.9%) | 30  (19.4%) | <0.001** |
| ***Trichuris*** | **21/243(8.64%)** | 0  (0%) | 16  (76.2%) | 5  (23.8%) | <0.001** |
| **Mites** | **243/243(100%)** | 71  (28.5%) | 119  (48%) | 53  (23.5%) | <0.001** |
| **Autumn** | | | | | |
| ***Cryptosporidium*** | **195/197(98.98%)** | 59  (30.3%) | 112  (57.4%) | 24  (12.3%) | <0.001** |
| ***Entamoeba*** | **123/197(62.44%)** | 13  (10.2%) | 102  (83.2%) | 8  (6.6%) | <0.001** |
| ***Balantidium*** | **63/197(31.98%)** | 9  (13.9%) | 53  (84.5%) | 1  (1.6%) | <0.001** |
| **Trichostrongylidae** | **100/197(50.76%)** | 25  (24.5%) | 75  (75.5%) | 0  (0%) | <0.001** |
| ***Trichuris*** | **80/197(40.61%)** | 2  (1.9%) | 78  (98.1%) | 0  (0%) | <0.001** |
| **Mites** | **179/197(90.86%)** | 60  (33.4%) | 108  (60.4%) | 11  (6.2%) | <0.001** |
| **Spring** | | | | | |
| ***Cryptosporidium*** | **222/226(98.2%)** | 94  (42.4%) | 86  (38.8%) | 42  (18.8%) | <0.001** |
| ***Giardia*** | **3/226(1.33%)** | 0  (0%) | 3  (100%) | 0  (0%) | - |
| ***Entamoeba*** | **175/226(77.43%)** | 45  (26%) | 85  (48.5%) | 45  (25.5%) | <0.001** |
| ***Balantidium*** | **122/226(53.98%)** | 20  (16.5%) | 72  (59.1%) | 30  (24.4%) | <0.001** |
| **Trichostrongylidae** | **63/226(27.88%)** | 12  (19%) | 51  (81%) | 0  (0%) | <0.001** |
| ***Trichuris*** | **32/226(14.16%)** | 1  (3.2%) | 31  (96.8%) | 0  (0%) | <0.001** |
| **Mites** | **215/226(95.1%)** | 94  (43.6%) | 84  (39.2%) | 37  (17.2%) | <0.001** |
| **Winter** | | | | | |
| ***Cryptosporidium*** | **86/106(81.1%)** | **30**  **(34.5%)** | **50**  **(58.1%)** | **6**  **(7.4%)** | **<0.001**** |
| ***Entamoeba*** | **24/106(22.64%)** | 0  (0%) | 24  (100%) | 0  (0%) | - |
| ***Balantidium*** | **9/106(8.49%)** | 0  (0%) | 9  (100%) | 0  (0%) | - |
| **Trichostrongylidae** | **29/106(27.36%)** | 0  (0%) | 29  (100%) | 0  (0%) | - |
| ***Trichuris*** | **4/106(3.77%)** | 0  (0%) | 4  (100%) | 0  (0%) | - |
| **Mites** | **82/106(77.36%)** | 32  (38.7%) | 48  (58.9%) | 2  (2.5%) | <0.001** |

**Table S2: Seasonal distribution of parasite infestation of *Musca sorbens* based on sedimentation-floatation technique and microscopic examination.**

|  | **Positive pools/ Total (%)** | **Site A** | **Site B** | **Site C** | ***P. value*** |
| --- | --- | --- | --- | --- | --- |
| **Summer** | | | | | |
| ***Cryptosporidium*** | **6/6(100%)** | 4  (66.7%) | 2  (33.3%) | 0  (0%) | 0.082 |
| ***Giardia*** | **1/6(16.67%)** | 0  (0%) | 1  (100%) | 0  (0%) | - |
| ***Entamoeba*** | **6/6(100%)** | 4  (66.7%) | 2  (33.3%) | 0  (0%) | 0.007** |
| ***Balantidium*** | **3/6(50%)** | 1  (33.3%) | 2  (66.7%) | 0  (0%) | 0.004** |
| **Trichostrongylidae** | **1/6(16.67%)** | 1  (100%) | 0  (0%) | 0  (0%) | - |
| ***Trichuris*** | **2/6(33.3%)** | 0  (0%) | 2  (100%) | 0  (0%) | - |
| **Mites** | **6/6(100%)** | 4  (66.7%) | 2  (33.3%) | 0  (0%) | 0.082 |
| **Autumn** | | | | | |
| ***Cryptosporidium*** | **2/2(100%)** | 1  (50%) | 1  (50%) | 0  (0%) | 0.182 |
| ***Giardia*** | **1/2(50%)** | 0  (0%) | 1  (100%) | 0  (0%) | - |
| ***Entamoeba*** | **2/2(100%)** | 1  (50%) | 1  (50%) | 0  (0%) | 0.579 |
| ***Balantidium*** | **1/2(50%)** | 0  (0%) | 1  (100%) | 0  (0%) | 1.000 |
| **Trichostrongylidae** | **2/2(100%)** | 1  (50%) | 1  (50%) | 0  (0%) | 0.617 |
| ***Trichuris*** | **1/2(50%)** | 0  (0%) | 1  (100%) | 0  (0%) | - |
| **Mites** | **2/2(100%)** | 1  (50%) | 1  (50%) | 0  (0%) | 0.182 |

**Table S3: Seasonal distribution of parasite infestation of *Stomoxys calcitrans* based on sedimentation-floatation technique and microscopic examination.**

|  | **Positive pools/ Total (%)** | **Site A** | **Site B** | **Site C** | ***P. value*** |
| --- | --- | --- | --- | --- | --- |
| **Summer** | | | | | |
| ***Cryptosporidium*** | **8/8(100%)** | 1  (12.5%) | 1  (12.5%) | 6  (75%) | <0.001** |
| ***Giardia*** | **1/8(12.5%)** | 0  (0%) | 1  (100%) | 0  (0%) | - |
| ***Entamoeba*** | **7/8(87.5%)** | 1  (14.29%) | 1  (14.29%) | 5  (71.4%) | <0.001** |
| ***Balantidium*** | **5/8(62.5%)** | 0  (0%) | 1  (20%) | 4  (80%) | <0.001** |
| **Trichostrongylidae** | **1/8(12.5%)** | 0  (0%) | 1  (100%) | 0  (0%) | - |
| ***Trichuris*** | **1/8(12.5%)** | 0  (0%) | 1  (100%) | 0  (0%) | - |
| **Mites** | **7/8(87.5%)** | 1  (14.29%) | 1  (14.29%) | 5  (71.43%) | <0.001** |
| **Autumn** | | | | | |
| ***Cryptosporidium*** | **4/5(80%)** | 1  (25%) | 1  (25%) | 2  (50%) | <0.001** |
| ***Giardia*** | **1/5(20%)** | 0  (0%) | 1  (100%) | 0  (0%) | - |
| ***Entamoeba*** | **2/5(40%)** | 1  (50%) | 1  (50%) | 0  (0%) | 0.617 |
| ***Balantidium*** | **2/5(40%)** | 1  (50%) | 1  (50%) | 0  (0%) | 0.617 |
| **Trichostrongylidae** | **2/5(40%)** | 1  (50%) | 1  (50%) | 0  (0%) | 0.617 |
| ***Trichuris*** | **1/5(20%)** | 0  (0%) | 1  (100%) | 0  (0%) | - |
| **Mites** | **5/5(100%)** | 1  (20%) | 1  (20%) | 3  (60%) | <0.001** |
| **Spring** | | | | | |
| ***Cryptosporidium*** | **5/6(83.3%)** | 1  (20%) | 1  (20%) | 3  (60%) | <0.001** |
| ***Entamoeba*** | **6/6(100%)** | 1  (16.7%) | 1  (16.7%) | 4  (66.7%) | <0.001** |
| ***Balantidium*** | **5/6(83.3%)** | 1  (20%) | 1  (20%) | 3  (60%) | <0.001** |
| **Trichostrongylidae** | **1/6(16.67%)** | 0  (0%) | 1  (100%) | 0  (0%) | - |
| ***Trichuris*** | **1/6(16.67%)** | 0  (0%) | 1  (100%) | 0  (0%) | - |
| **Mites** | **6/6(100%)** | 1  (16.7%) | 1  (16.7%) | 4  (66.7%) | <0.001** |
| **Winter** | | | | | |
| ***Cryptosporidium*** | **2/3(66.67%)** | 1  (50%) | 1  (50%) | 0  (0%) | 0.371 |
| ***Entamoeba*** | **1/3(33.3%)** | 0  (0%) | 1  (100%) | 0  (0%) | **-** |
| ***Balantidium*** | **1/3(33.3%)** | 0  (0%) | 1  (100%) | 0  (0%) | **-** |
| **Trichostrongylidae** | **1/3(33.3%)** | 0  (0%) | 1  (100%) | 0  (0%) | **-** |
| **Mites** | **1/3(33.3%)** | 0  (0%) | 1  (100%) | 0  (0%) | **-** |

**Table S4: Seasonal distribution of parasite infestation of *Borborillus vitripennis* based on sedimentation-floatation technique and microscopic examination.**

|  | **Positive pools/ Total (%)** | **Site A** | **Site B** | **Site C** | ***P. value*** |
| --- | --- | --- | --- | --- | --- |
| **Summer** | | | | | |
| ***Cryptosporidium*** | **85/98(86.73%)** | 32  (37.6%) | 34  (40%) | 19  (22.4%) | <0.001** |
| ***Giardia*** | **2/98(2.04%)** | 0  (0%) | 2  (100%) | 0  (0%) | - |
| ***Entamoeba*** | **49/98(50%)** | 17  (34.4%) | 25  (52.1%) | 7  (13.5%) | <0.001** |
| ***Balantidium*** | **36/98(36.73%)** | 4  (11.11%) | 27  (75%) | 5  (13.9%) | <0.001** |
| ***Trichuris*** | **8/98(8.16%)** | 0  (0%) | 8  (100%) | 0  (0%) | - |
| **Autumn** | | | | | |
| ***Cryptosporidium*** | **46/55(83.64%)** | 12  (26.3%) | 27  (59.2%) | 7  (14.5%) | <0.001** |
| ***Giardia*** | **2/55(3.64%)** | 0  (0%) | 2  (100%) | 0  (0%) | - |
| ***Entamoeba*** | **25/55(45.45%)** | 9  (36.2%) | 12  (46.8%) | 4  (17%) | <0.001** |
| ***Balantidium*** | **25/55(45.45%)** | 6  (24.5%) | 17  (69.4%) | 2  (6.1%) | <0.001** |
| **Trichostrongylidae** | **38/55(69.09%)** | 11  (28%) | 27  (72%) | 0  (0%) | <0.001** |
| ***Trichuris*** | **10/55(18.18%)** | 4  (40%) | 6  (60%) | 0  (0%) | 0.057 |
| **Spring** | | | | | |
| ***Cryptosporidium*** | **78/94(82.98%)** | 30  (38.5%) | 38  (48.7%) | 10  (12.8%) | <0.001** |
| ***Entamoeba*** | **35/94(37.23%)** | 13  (38.2%) | 16  (45.6%) | 6  (16.2%) | <0.001** |
| ***Balantidium*** | **32/94(34.04%)** | 8  (25%) | 20  (62.5%) | 4  (12.5%) | <0.001** |
| **Trichostrongylidae** | **47/94(50%)** | 9  (19.1%) | 38  (80.9%) | 0  (0%) | <0.001** |
| ***Trichuris*** | **11/94(11.7%)** | 3  (28.6%) | 8  (71.4%) | 0  (0%) | 0.276 |
| **Winter** | | | | | |
| ***Cryptosporidium*** | **29/45(64.44%)** | 3  (10.3%) | 25  (86.2%) | 1  (3.4%) | <0.001** |
| ***Entamoeba*** | **5/45(11.11%)** | 3  (66.7%) | 0  (0%) | 2  (33.3%) | 0.037* |
| ***Balantidium*** | **4/45(8.89%)** | 0  (0%) | 4  (100%) | 0  (0%) | - |
| **Trichostrongylidae** | **8/45(17.78%)** | 0  (0%) | 8  (100%) | 0  (0%) | - |
| ***Trichuris*** | **2/45(4.44%)** | 0  (0%) | 2  (100%) | 0  (0%) | - |

**Table S5: Seasonal distribution of parasite infestation of *Fannia canicularis* based on sedimentation-floatation technique and microscopic examination.**

|  | **Positive pools/ Total (%)** | **Site A** | **Site B** | **Site C** | ***P. value*** |
| --- | --- | --- | --- | --- | --- |
| **Summer** | | | | | |
| ***Cryptosporidium*** | **37/39(94.87%)** | 14  (38.4%) | 21  (56.2%) | 2  (5.4%) | <0.001** |
| ***Giardia*** | **1/39(2.56%)** | 0  (0%) | 1  (100%) | 0  (0%) | - |
| ***Entamoeba*** | **29/39(74.36%)** | 7  (24.14%) | 21  (72.2%) | 1  (4.9%) | <0.001** |
| ***Balantidium*** | **12/39(30.77%)** | 3  (22.1%) | 8  (70.8%) | 1  (7.1%) | <0.001** |
| ***Trichuris*** | **4/39(10.26%)** | 1  (25%) | 3  (75%) | 0  (0%) | 0.002** |
| **Mites** | **12/39(30.77%)** | 4  (31.8%) | 6  (54.5%) | 2  (13.6%) | <0.001** |
| **Autumn** | | | | | |
| ***Cryptosporidium*** | **23/23(100%)** | 9  (39.13%) | 13  (56.5%) | 1  (4.4%) | <0.001** |
| ***Entamoeba*** | **14/23(60.87%)** | 4  (29%) | 9  (64.3%) | 1  (5.8%) | <0.001** |
| ***Balantidium*** | **6/23(26.09%)** | 1  (16.7%) | 4  (66.7%) | 1  (16.7%) | <0.001** |
| **Trichostrongylidae** | **18/23(78.26%)** | 6  (33.3%) | 12  (64.2%) | 0  (0%) | 0.001** |
| ***Trichuris*** | **3/23(13.04%)** | 1  (33.3%) | 2  (66.7%) | 0  (0%) | 0.211 |
| **Mites** | **6/23(26.09%)** | 2  (40%) | 2  (40%) | 2  (20%) | 0.397 |
| **Spring** | | | | | |
| ***Cryptosporidium*** | **34/35(97.14%)** | 14  (41.3%) | 18  (53.2%) | 2  (5.5%) | <0.001** |
| ***Entamoeba*** | **17/35(48.57%)** | 5  (29.4%) | 11  (64.7%) | 1  (5.88%) | <0.001** |
| ***Balantidium*** | **8/35(22.86%)** | 2  (25%) | 5  (62.5%) | 1  (12.5%) | <0.001** |
| **Trichostrongylidae** | **9/35(25.7%)** | 3  (32.3%) | 6  (67.7%) | 0  (0%) | 0.001** |
| ***Trichuris*** | **3/35(8.57%)** | 1  (33.3%) | 2  (66.7%) | 0  (0%) | 0.031* |
| **Mites** | **10/35(28.57%)** | 4  (40%) | 5  (50%) | 1  (10%) | <0.001** |
| **Winter** | | | | | |
| ***Cryptosporidium*** | **17/19(89.47%)** | 6  (35.29%) | 11  (64.7%) | 0  (0%) | <0.001** |
| ***Entamoeba*** | **2/19(10.53%)** | 1  (50%) | 1  (50%) | 0  (0%) | 0.423 |
| ***Balantidium*** | **2/19(10.53%)** | 1  (66.7%) | 0  (0%) | 1  (33.3%) | 0.683 |
| **Mites** | **3/19(15.79%)** | 1  (33.3%) | 1  (33.3%) | 1  (33.3%) | 0.472 |

**Table S6: Seasonal distribution of parasite infestation of *Sepsis punctum* based on sedimentation-floatation technique and microscopic examination.**

|  | **Positive pools/ Total (%)** | **Site A** | **Site B** | **Site C** | ***P. value*** |
| --- | --- | --- | --- | --- | --- |
| **Summer** | | | | | |
| ***Cryptosporidium*** | **19/19(100%)** | 7  (37.3%) | 10  (54.2%) | 2  (8.5%) | <0.001** |
| ***Giardia*** | **1/19(5.26%)** | 0  (0%) | 1  (100%) | 0  (0%) | - |
| ***Entamoeba*** | **14/19(73.68%)** | 6  (42%) | 7  (50.4%) | 1  (7.6%) | <0.001** |
| ***Balantidium*** | **9/19(47.37%)** | 4  (45.8%) | 4  (48.2%) | 1  (6%) | <0.001** |
| ***Trichuris*** | **3/19(15.79%)** | 1  (33.3%) | 1  (33.3%) | 1  (33.3%) | 0.296 |
| **Autumn** | | | | | |
| ***Cryptosporidium*** | **9/11(81.82%)** | 4  (44.45%) | 4  (44.45%) | 1  (11.1%) | <0.001** |
| ***Entamoeba*** | **6/11(54.55%)** | 2  (33.3%) | 3  (50%) | 1  (16.7%) | <0.001** |
| ***Balantidium*** | **4/11(36.36%)** | 2  (50%) | 2  (50%) | 0  (0%) | 0.627 |
| **Trichostrongylidae** | **11/11(100%)** | 3  (27.3%) | 8  (72.7%) | 0  (0%) | <0.001** |
| ***Trichuris*** | **2/11(18.18%)** | 1  (50%) | 1  (50%) | 0  (0%) | 0.724 |
| **Spring** | | | | | |
| ***Cryptosporidium*** | **15/16(93.75%)** | 5  (33.3%) | 9  (60%) | 1  (6.67%) | <0.001** |
| ***Giardia*** | **1/16(6.25%)** | 0  (0%) | 1  (100%) | 0  (0%) | - |
| ***Entamoeba*** | **8/16(50%)** | 3  (37.5%) | 4  (50%) | 1  (12.5%) | <0.001** |
| ***Balantidium*** | **7/16(43.75%)** | 3  (42.86%) | 3  (42.86%) | 1  (14.29%) | <0.001** |
| **Trichostrongylidae** | **5/16(31.25%)** | 1  (25.5%) | 4  (74.5%) | 0  (0%) | 0.001** |
| ***Trichuris*** | **2/16(12.5%)** | 1  (50%) | 1  (50%) | 0  (0%) | 0.547 |
| **Winter** | | | | | |
| ***Cryptosporidium*** | **7/10(70%)** | 2  (28.57%) | 5  (71.4%) | 0  (0%) | <0.001** |
| ***Entamoeba*** | **2/10(20%)** | 1  (50%) | 1  (50%) | 0  (0%) | 0.752 |
| ***Balantidium*** | **2/10(20%)** | 1  (50%) | 1  (50%) | 0  (0%) | 0.724 |
| ***Trichuris*** | **2/10(20%)** | 1  (50%) | 1  (50%) | 0  (0%) | 1.000 |

**Table S7: Seasonal distribution of parasite infestation of *Meroplius minutus* based on sedimentation-floatation technique and microscopic examination.**

|  | **Positive pools/Total (%)** | **Site A** | | **Site B** | **Site C** | ***P. value*** |
| --- | --- | --- | --- | --- | --- | --- |
| **Summer** | | | | | | |
| ***Cryptosporidium*** | **4/4(100%)** | 1  (25%) | | 1  (25%) | 2  (50%) | 0.067 |
| ***Giardia*** | **1/4(25%)** | 0  (0%) | | 1  (100%) | 0  (0%) | - |
| ***Entamoeba*** | **3/4(75%)** | 1  (33.3%) | | 1  (33.3%) | 1  (33.3%) | 0.422 |
| ***Balantidium*** | **2/4(50%)** | 0  (0%) | | 1  (50%) | 1  (50%) | 0.752 |
| ***Trichuris*** | **1/4(25%)** | 0  (0%) | | 1  (100%) | 0  (0%) | - |
| **Autumn** | | | | | | |
| ***Cryptosporidium*** | **3/3(100%)** | 1  (33.3%) | | 1  (33.3%) | 1  (33.3%) |  |
| ***Giardia*** | **1/3(33.3%)** | 0  (0%) | | 1  (100%) | 0  (0%) | - |
| ***Entamoeba*** | **3/3(100%)** | 1  (33.3%) | | 1  (33.3%) | 1  (33.3%) |  |
| ***Balantidium*** | **3/3(100%)** | 1  (33.3%) | | 1  (33.3%) | 1  (33.3%) |  |
| **Trichostrongylidae** | **2/3(66.67%)** | 1  (50%) | | 1  (50%) | 0  (0%) |  |
| ***Trichuris*** | **1/3(33.3%)** | 0  (0%) | | 1  (100%) | 0  (0%) | - |
| **Mites** | **3/3(100%)** | 1  (33.3%) | | 1  (33.3%) | 1  (33.3%) |  |
| **Spring** | | | | | | |
| ***Cryptosporidium*** | **4/5(80%)** | 1  (25%) | | 1  (25%) | 2  (50%) | 0.267 |
| ***Giardia*** | **1/5(20%)** | 0  (0%) | | 1  (100%) | 0  (0%) | - |
| ***Entamoeba*** | **3/5(60%)** | 1  (33.3%) | | 1  (33.3%) | 1  (33.3%) | 1.000 |
| ***Balantidium*** | **3/5(60%)** | 1  (33.3%) | | 1  (33.3%) | 1  (33.3%) | 0.449 |
| **Trichostrongylidae** | **2/5(40%)** | 1  (50%) | | 1  (50%) | 0  (0%) | 0.450 |
| ***Trichuris*** | **1/5(20%)** | 0  (0%) | | 1  (100%) | 0  (0%) | - |
| **Mites** | **3/5(60%)** | 1  (33.3%) | 1  (33.3%) | | 1  (33.3%) | 0.216 |
| **Winter** | | | | | | |
| ***Cryptosporidium*** | **3/3(100%)** | 1  (33.3%) | 1  (33.3%) | | 1  (33.3%) | 0.223 |
| ***Entamoeba*** | **1/3(33.3%)** | 0  (0%) | 1  (100%) | | 0  (0%) | - |
| ***Balantidium*** | **1/3(33.3%)** | 0  (0%) | 1  (100%) | | 0  (0%) | - |
| **Trichostrongylidae** | **1/3(33.3%)** | 0  (0%) | 1  (100%) | | 0  (0%) | - |
| ***Trichuris*** | **1/3(33.3%)** | 0  (0%) | 1  (100%) | | 0  (0%) | - |
| **Mites** | **2/3(66.67%)** | 1  (50%) | 1  (50%) | | 0  (0%) | 0.371 |

**Table S8: Seasonal distribution of parasite infestation of *Physiphora alceae* based on sedimentation-floatation technique and microscopic examination.**

|  | **Positive pools/ Total (%)** | **Site A** | **Site B** | **Site C** | ***P. value*** |
| --- | --- | --- | --- | --- | --- |
| **Summer** | | | | | |
| ***Cryptosporidium*** | **6/6(100%)** | 4  (66.7%) | 1  (16.7%) | 1  (16.7%) | <0.001** |
| ***Giardia*** | **1/6(16.67%)** | 0  (0%) | 1  (100%) | 0  (0%) | - |
| ***Entamoeba*** | **4/6(66.67%)** | 2  (50%) | 1  (25%) | 1  (25%) | 0.001** |
| ***Balantidium*** | **3/6(50%)** | 1  (33.3%) | 1  (33.3%) | 1  (33.3%) | 0.030* |
| ***Trichuris*** | **1/6(16.67%)** | 0  (0%) | 1  (100%) | 0  (0%) | - |
| **Mites** | **6/6(100%)** | 4  (66.7%) | 1  (16.7%) | 1  (16.7%) | <0.001** |
| **Autumn** | | | | | |
| ***Cryptosporidium*** | **2/3(66.67%)** | 1  (50%) | 1  (50%) | 0  (0%) | 0.480 |
| ***Giardia*** | **1/3(33.3%)** | 0  (0%) | 1  (100%) | 0  (0%) | - |
| ***Entamoeba*** | **2/3(66.67%)** | 1  (50%) | 1  (50%) | 0  (0%) | 0.480 |
| ***Balantidium*** | **3/3(100%)** | 1  (33.3%) | 1  (33.3%) | 1  (33.3%) | 1.000 |
| **Trichostrongylidae** | **2/3(66.67%)** | 1  (50%) | 1  (50%) | 0  (0%) | 0.480 |
| **Mites** | **2/3(66.67%)** | 0  (0%) | 1  (50%) | 1  (50%) | 0.480 |
| **Spring** | | | | | |
| ***Cryptosporidium*** | **7/7(100%)** | 5  (71.4%) | 1  (14.28%) | 1  (14.28%) | <0.001** |
| ***Giardia*** | **1/7(14.29%)** | 0  (0%) | 1  (100%) | 0  (0%) | - |
| ***Entamoeba*** | **5/7(71.43%)** | 3  (60%) | 1  (20%) | 1  (20%) | <0.001** |
| ***Balantidium*** | **3/7(42.86%)** | 1  (33.3%) | 1  (33.3%) | 1  (33.3%) | 0.529 |
| **Trichostrongylidae** | **2/7(28.57%)** | 1  (50%) | 1  (50%) | 0  (0%) | 1.000 |
| ***Trichuris*** | **1/7(14.29%)** | 0  (0%) | 1  (100%) | 0  (0%) | - |
| **Mites** | **7/7(100%)** | 5  (71.4%) | 1  (14.28%) | 1  (14.28%) | <0.001** |
| **Winter** | | | | | |
| ***Cryptosporidium*** | **3/4(75%)** | 2  (66.7%) | 1  (33.3%) | 0  (0%) | 0.014* |
| ***Entamoeba*** | **2/4(50%)** | 1  (50%) | 1  (50%) | 0  (0%) | 1.000 |
| ***Balantidium*** | **2/4(50%)** | 1  (50%) | 1  (50%) | 0  (0%) | 1.000 |
| **Trichostrongylidae** | **1/4(25%)** | 1(100%) | 0(0%) | 0  (0%) | - |
| **Mites** | **4/4(100%)** | 2  (50%) | 1  (25%) | 1  (25%) | <0.001** |

**Table S9: Seasonal distribution of midge infestations of different fly species collected from site B based on sedimentation-floatation technique and microscopic examination.**

| **Fly family** | **Fly species** | **Total**  **Pools in Site B** | **Total positive fly pools for Midges** | **Summer** | **Autumn** | **Spring** | ***P*. value** |
| --- | --- | --- | --- | --- | --- | --- | --- |
| **Muscidae** | *Musca domestica* | 367 | 132(42.5%) | 22 | 55 | 57 | <0.001** |
|  | *Musca sorbens* | 3 | 3(0.97%) | 1 | 1 | 1 | - |
| **Sphaeroceridae** | *Borborillus vitripennis* | 124 | 98(16.5%) | 12 | 30 | 56 | <0.001** |
| **Fanniidae** | *Fannia canicularis* | 62 | 52(8.8%) | 9 | 21 | 22 | <0.001** |
| **Sepsidae** | *Sepsis punctum* | 28 | 16(2.7%) | 4 | 5 | 7 | <0.042** |
|  | *Meroplius minutus* | 3 | 3(0.97%) | 1 | 1 | 1 | - |
| **Ulidiidae** | *Physiphora alceae* | 4 | 3(0.97%) | 1 | 1 | 1 | - |
| **Calliphoridae** | *Calliphora vicina* | 1 | 1(0.17%) | 0 | 1 | 0 | - |
|  | *Chrysomya megacephala* | 2 | 1(0.17%) | 0 | 0 | 1 | - |
|  | *Lucilia sericata* | 1 | 1(0.17%) | 1 | 0 | 0 | - |
| **Total** |  | 595 | 310 (52.1%) |  |  |  |  |
